# Myxoid Liposarcoma-Associated EWSR1-DDIT3 Selectively Represses Osteoblastic and Chondrocytic Transcription in Multipotent Mesenchymal Cells

**DOI:** 10.1371/journal.pone.0036682

**Published:** 2012-05-03

**Authors:** Kayo Suzuki, Yoshito Matsui, Mami Higashimoto, Yoshiharu Kawaguchi, Shoji Seki, Hiraku Motomura, Takeshi Hori, Yasuhito Yahara, Masahiko Kanamori, Tomoatsu Kimura

**Affiliations:** 1 Department of Orthopaedic Surgery, Faculty of Medicine, University of Toyama, Toyama, Japan; 2 Department of Human Science, Faculty of Medicine, University of Toyama, Toyama, Japan; The National Institute of Diabetes and Digestive and Kidney Diseases, United States of America

## Abstract

**Background:**

Liposarcomas are the most common class of soft tissue sarcomas, and myxoid liposarcoma is the second most common liposarcoma. EWSR1-DDIT3 is a chimeric fusion protein generated by the myxoid liposarcoma-specific chromosomal translocation t(12;22)(q13;q12). Current studies indicate that multipotent mesenchymal cells are the origin of sarcomas. The mechanism whereby EWSR1-DDIT3 contributes to the phenotypic selection of target cells during oncogenic transformation remains to be elucidated.

**Methodology/Principal Findings:**

Reporter assays showed that the EWSR1-DDIT3 myxoid liposarcoma fusion protein, but not its wild-type counterparts EWSR1 and DDIT3, selectively repressed the transcriptional activity of cell lineage-specific marker genes in multipotent mesenchymal C3H10T1/2 cells. Specifically, the osteoblastic marker Opn promoter and chondrocytic marker Col11a2 promoter were repressed, while the adipocytic marker Ppar-γ2 promoter was not affected. Mutation analyses, transient ChIP assays, and treatment of cells with trichostatin A (a potent inhibitor of histone deacetylases) or 5-Aza-2′-deoxycytidine (a methylation-resistant cytosine homolog) revealed the possible molecular mechanisms underlying the above-mentioned selective transcriptional repression. The first is a genetic action of the EWSR1-DDIT3 fusion protein, which results in binding to the functional C/EBP site within Opn and Col11a2 promoters through interaction of its DNA-binding domain and subsequent interference with endogenous C/EBPβ function. Another possible mechanism is an epigenetic action of EWSR1-DDIT3, which enhances histone deacetylation, DNA methylation, and histone H3K9 trimethylation at the transcriptional repression site. We hypothesize that EWSR1-DDIT3-mediated transcriptional regulation may modulate the target cell lineage through target gene-specific genetic and epigenetic conversions.

**Conclusions/Significance:**

This study elucidates the molecular mechanisms underlying EWSR1-DDIT3 fusion protein-mediated phenotypic selection of putative target multipotent mesenchymal cells during myxoid liposarcoma development. A better understanding of this process is fundamental to the elucidation of possible direct lineage reprogramming in oncogenic sarcoma transformation mediated by fusion proteins.

## Introduction

Sarcoma is the collective name for non-epithelial, non-hematopoietic malignant tumors that arise from the embryonic mesoderm. Several sarcomas have specific chromosomal translocations and resultant fusion genes [1]. In certain subsets of sarcomas that are believed to originate from multipotent mesenchymal cells, a specific sarcoma phenotype may manifest through transcriptional regulation by specific fusion proteins, modulating target cell lineages [2]–[5].

Liposarcomas are the most common class of soft tissue sarcomas and are divided into separate clinicopathological entities with distinctive morphological spectra and associated genetic changes [6]. Myxoid liposarcoma (MLS) denotes one such entity and is the second most common liposarcoma after well-differentiated liposarcoma [7]. A significant proportion of MLS has a cytogenetic hallmark of chromosomal translocation, t(12;16)(q13;p11). This translocation leads to fusion of translocated in liposarcoma (TLS; also known as fused in sarcoma, FUS) and DNA damage-inducible transcript 3 (DDIT3; also known as CCAAT/enhancer-binding protein (C/EBP) homologous protein, CHOP; originally named as growth arrest- and DNA damage- inducible gene 153, GADD153) genes, resulting in the production of the TLS-DDIT3 fusion protein [8]–[13].

In other subset of MLS, a variant chromosomal translocation, t(12;22)(q13;q12), results in fusion of Ewing's sarcoma (EWSR1) and DDIT3 genes [10], [11], [14]–[16]. However, the function of the resultant fusion protein EWSR1-DDIT3 during oncogenic transformation is not clear. If MLS originates from multipotent mesenchymal cells, EWSR1-DDIT3 may act as an aberrant transcription factor and affect the phenotypic selection of uncommitted target cells [17], [18]. To test this hypothesis, we analyzed whether EWSR1-DDIT3 affected the transcriptional potential of lineage-specific marker genes in mouse multipotent mesenchymal C3H10T1/2 cells. The osteopontin (Opn), alpha 2 chain of type XI collagen (Col11a2), and peroxisome proliferator-activated receptor-gamma (Ppar-γ) genes were selected to represent expression of osteoblastic, chondrocytic, and adipocytic phenotypes, respectively. We found that EWSR1-DDIT3 repressed the promoter activity of Opn and Col11a2 but not that of Ppar-γ2, and we further explored the potential molecular mechanisms underlying this selective transcriptional repression.

## Results

### Innate mouse multipotent mesenchymal C3H10T1/2 cells expressed Opn, Col11a2, and Ppar-γ mRNA transcripts

**Figure 1:**
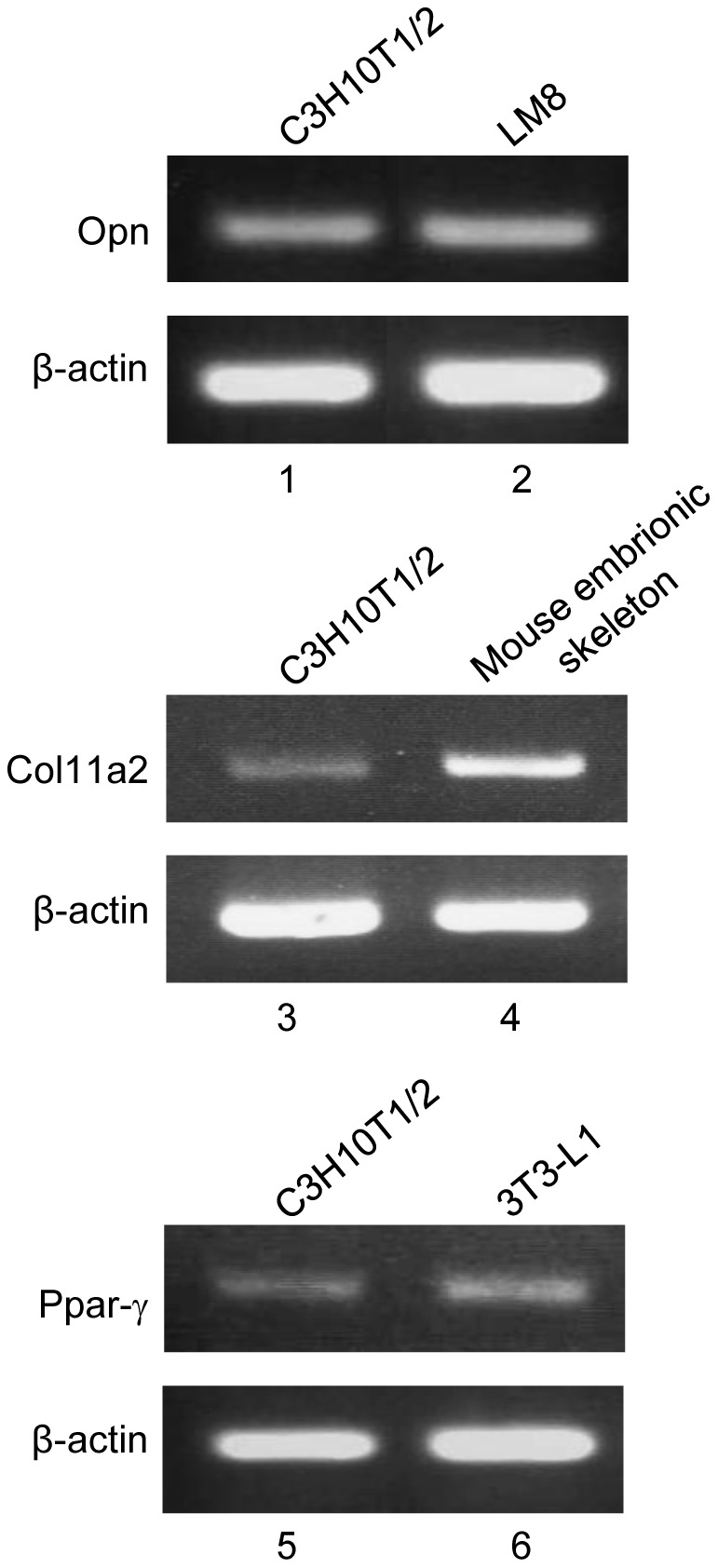
Innate mouse multipotent mesenchymal C3H10T1/2 cells expressed multiple lineage-specific marker genes. RT-PCR analysis detected osteoblastic marker Opn (lane 1), chondrocytic marker Col11a2 (lane 3), and adipocytic marker Ppar-γ (lane 5) mRNA transcripts in C3H10T1/2 cells. Mouse osteosarcoma cell line LM8 (lane 2), mouse embryonic skeleton cells (lane 4), and mouse preadipocytic cell line 3T3-L1 (lane 6) were analyzed as positive controls for Opn, Col11a2, and Ppar-γ gene expression, respectively. The β-actin transcript level served as a loading control for each reaction.

Opn is a phosphorylated glycoprotein originally isolated from bone [19] and is a marker for the osteoblastic cell phenotype [20]. Type XI collagen is almost exclusively found in the cartilage. Col11a2 gene encodes its alpha 2 chain [21], and Col1la2 expression is a marker for the chondrocytic cell phenotype [22]–[24]. Ppar-γ is a well-known master regulator of adipogenesis [25]. Two isoforms of Ppar-γ, Ppar-γ1 and Ppar-γ2, are generated by alternative splicing. Ppar-γ2 is more closely related to the adipocytic cell phenotype [26]. Reverse transcription-polymerase chain reaction (RT-PCR) analysis demonstrated that mRNA transcripts for Opn, Col11a2, and Ppar-γ genes were detectable in innate C3H10T1/2 cells (Figure 1). Thus, C3H10T1/2 cells simultaneously expressed multiple cell lineage-specific marker genes for osteoblastic, chondrocytic, and adipocytic phenotypes according to their multipotency [27]–[29].

### EWSR1-DDIT3 fusion protein, but not its wild-type counterparts EWSR1 and DDIT3, repressed the promoter activity of Opn and Col11a2 but not that of Ppar-γ2

**Figure 2:**
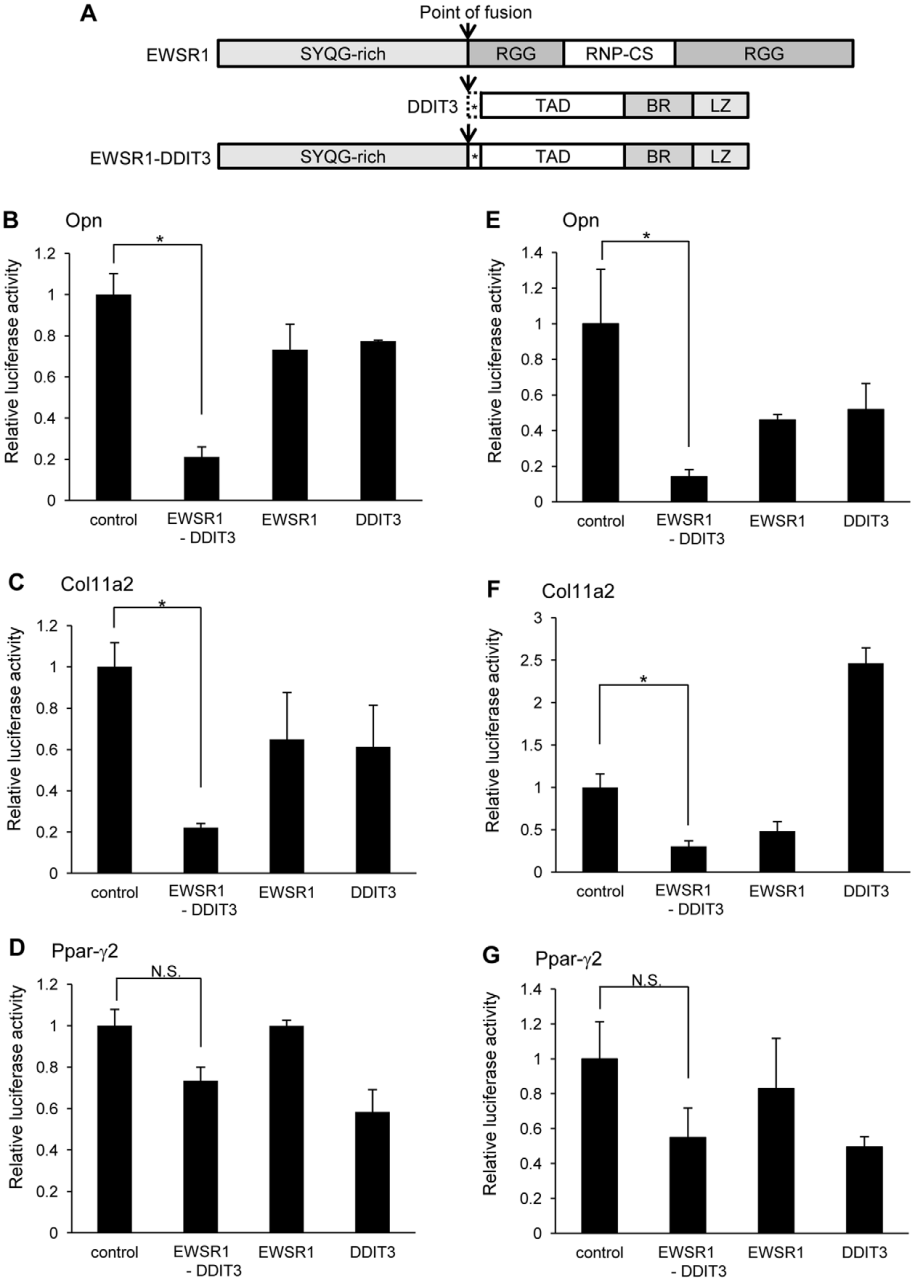
EWSR1-DDIT3 fusion protein selectively repressed promoter activities of lineage-specific marker genes in C3H10T1/2 cells as well as in hMSCs. (**A**) Schematic of domain structure of EWSR1, DDIT3, and EWSR1-DDIT3. SYQG-rich, Ser-Tyr-Gln-Gly-rich transactivating domain; RGG, regions with multiple Arg-Gly-Gly repeats; RNP-CS, ribonucleoprotein consensus sequence; TAD, transcriptional activation domain; BR, basic amino-acid-rich dimerization domain; LZ, leucine zipper DNA-binding domain. Asterisks (*) designate 27 amino acid residues originating from DNA sequences upstream of the translation start site of DDIT3 (broken line), which are translated after in-frame fusion to EWSR1. Arrows indicate points of fusion. (**B–D**) Effect of EWSR1-DDIT3, EWSR1, and DDIT3 expression vectors on the activities of Opn (**B**), Col11a2 (**C**), and Ppar-γ2 (**D**) promoter-luciferase constructs 48 h after transfection in C3H10T1/2 cells. EWSR1-DDIT3, but not its wild-type counterparts EWSR1 or DDIT3, repressed Opn and Col11a2 promoter activities. However, Ppar-γ2 promoter activity was not repressed. Transfection in duplicate was repeated at least three times, and the results are shown as averages ± SE. *p* values calculated by ANOVA were 0.001, 0.0159, and 0.005, for Opn, Col11a2, and Ppar-γ2, respectively. Asterisks (*) indicate statistical significance (*p*<0.05) following Tukey–Kramer post-hoc test. N.S., not significant. (**E–G**) Human bone marrow-derived mesenchymal stem cells (hMSCs) were purchased from Lonza Corporation, Walkersville, MD, USA, and cultured in MSC growth media (MSCGM-CD™ BulletKit™, Lonza) with penicillin (100 U/ml), streptomycin (100 µg/ml), and amphotericin B (0.25 µg/ml) under 5% CO_2_ at 37°C. Opn (**E**), Col11a2 (**F**), or Ppar-γ2 (**G**) promoter activity was analyzed from cell lysates extracted from cells cotransfected with pFLAG-CMV4 control, EWSR1-DDIT3, EWSR1, or DDIT3 48 h after transfection. These constructs were cotransfected with an internal control vector (Renilla). The luciferase activities were expressed as relative activity to that of the promoter-less reporter vector (pGL3 basic). Transfection in duplicate was repeated at least three times and the results are shown as average ± SE. *p* values calculated by ANOVA were 0.0445, <0.0001, and 0.2846, for Opn, Col11a2, and Ppar-γ2, respectively.

A report of successful transformation in the same cellular background, i.e., by induction of a single oncogenic event (specifically, induction of the EWSR1 fusion protein, EWSR1-FLI1) [30], encouraged us to analyze the molecular mechanism of sarcomagenesis caused by EWSR1-DDIT3 using C3H10T1/2 cells. To investigate whether the EWSR1-DDIT3 fusion protein influences transcription of Opn, Col11a2, or Ppar-γ2 genes, we first monitored the promoter activity of the −857/+91 fragment of the mouse Opn promoter-, −742/+380 fragment of the mouse Col11a2 promoter-, and −615/+64 fragment of the mouse Ppar-γ2 promoter-luciferase reporter constructs (pGL3-Opn, pGL3-Col11a2, and pGL3-Ppar-γ2, respectively) in C3H10T1/2 cells. Transient cotransfection experiments demonstrated that each promoter construct exhibited promoter activity when transfected with the empty vector, pFLAG-CMV4 control. Overexpressing EWSR1-DDIT3 under the control of a CMV promoter using the pFLAG-CMV4 EWSR1-DDIT3 expression vector significantly repressed Opn and Col11a2 promoter activities by 79% and 78%, respectively; however, overexpressing EWSR1 and DDIT3 did not (Figure 2A–C). On the other hand, Ppar-γ2 promoter activity was not significantly affected by EWSR1-DDIT3 or EWSR1 overexpression, but it was very mildly repressed by wild-type DDIT3 (Figure 2D). The latter finding is consistent with the inhibitory action of DDIT3 on the Ppar-γ2 promoter in rat osteosarcoma-derived UMR106 cells [31]. These data indicate that the EWSR1-DDIT3 fusion protein selectively represses transcription from osteoblastic and chondroblastic lineage marker genes, but not from an adipocytic lineage marker gene, in multipotent mesenchymal C3H10T1/2 cells. Further experiments using human mesenchymal stem cells (hMSCs, Lonza Corporation, Walkersville, MD, USA) [32], [33] also showed that the EWSR1-DDIT3 fusion protein selectively repressed transcription from Opn and Col11a2 promoters, but not from Ppar-γ2 promoter (Figure 2E–G). These observations suggested that the selective gene repression by the EWSR1-DDIT3 fusion protein may be more than an artifact dependent on the uniqueness of C3H10T1/2 cell line.

### Repressive action of EWSR1-DDIT3 on the transcriptional activity of Opn and Col11a2 genes required its intact leucine zipper domain

**Figure 3:**
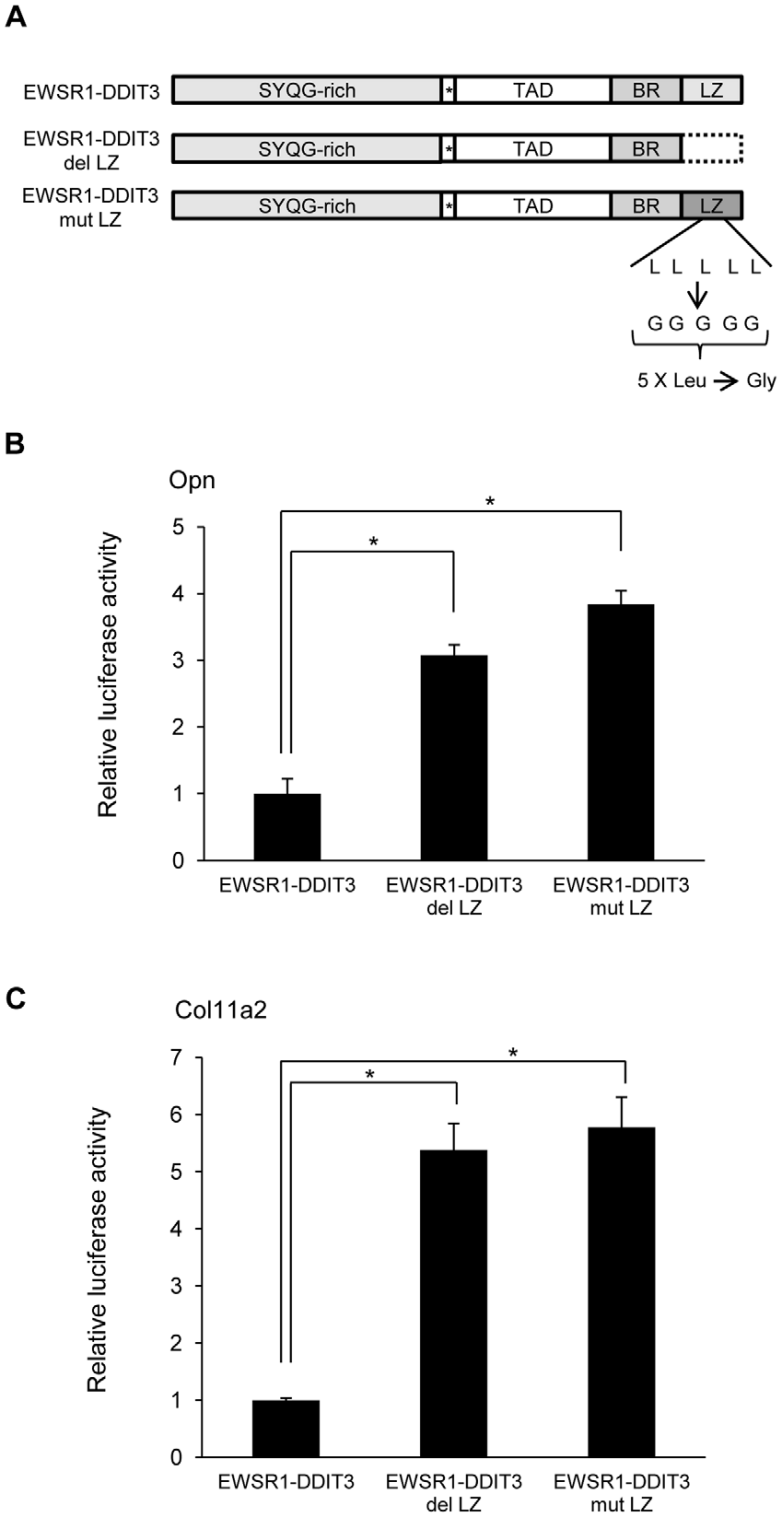
Intact LZ dimer forming domain was essential for EWSR1-DDIT3-mediated repression of Opn and Col11a2 promoter activities. (**A**) Schematic drawing of EWSR1-DDIT3 and two mutants. SYQG-rich, Ser-Tyr-Gln-Gly-rich transactivating domain; TAD, transcriptional activation domain; BR, basic amino-acid-rich dimerization domain; LZ, leucine zipper dimerforming domain. Asterisks (*) designate 27 amino acid residues originating from DNA sequences upstream of the translation start site of DDIT3. EWSR1-DDIT3 del LZ lacks the entire LZ composed of 38 C-terminal amino acid residues (broken line). In EWSR1-DDIT3 mut LZ, all five leucine residues in LZ are converted to glycine residues, as designated. (**B** and **C**) Effect of EWSR1-DDIT3 or two forms of mutant expression vectors on the activities of Opn (**B**) and Col11a2 (**C**) promoter-luciferase constructs 48 h after transfection in C3H10T1/2 cells. Opn and Col11a2 promoter activities were significantly increased by EWSR1-DDIT3 del LZ and EWSR1-DDIT3 mut LZ than by EWSR1-DDIT3. Transfection in duplicate was repeated at least three times, and the results are shown as averages ± SE. *p* values calculated by ANOVA were 0.0001 for Opn and 0.0003 for Col11a2. Asterisks (*) indicate statistical significance (*p*<0.05) following Tukey–Kramer post-hoc test.

Molecular composition of EWSR1-DDIT3 revealed that the C-terminal DDIT3 sequence contained a leucine zipper (LZ) dimer forming domain (Figure 3A). DDIT3 is a dimer forming transcription factor and it can not bind DNA as a monomer nor form dimers with itself. To test the possibility that EWSR1-DDIT3 affects Opn and Col11a2 promoters by binding to potential target sites within them, two forms of mutant EWSR1-DDIT3 expression vectors were generated. One mutant, EWSR1-DDIT3 del LZ lacked 38 amino acids at the C-terminal end, which contained the entire LZ domain. In another mutant, EWSR1-DDIT3 mut LZ, all five leucine residues within the dimer forming domain were mutated to glycine residues. Consequent cotransfection experiments in C3H10T1/2 cells documented that Opn and Col11a2 promoter activities were significantly increased by overexpression of EWSR1-DDIT3 del LZ or EWSR1-DDIT3 mut LZ than of EWSR1-DDIT3 (Figure 3B and 3C). These observations revealed that the repressive action of EWSR1-DDIT3 required its intact LZ domain for binding to potential target sites within each promoter.

### Function of potential C/EBP-binding sites within Opn and Col11a2 promoters in mouse multipotent mesenchymal C3H10T1/2 cells

**Figure 4:**
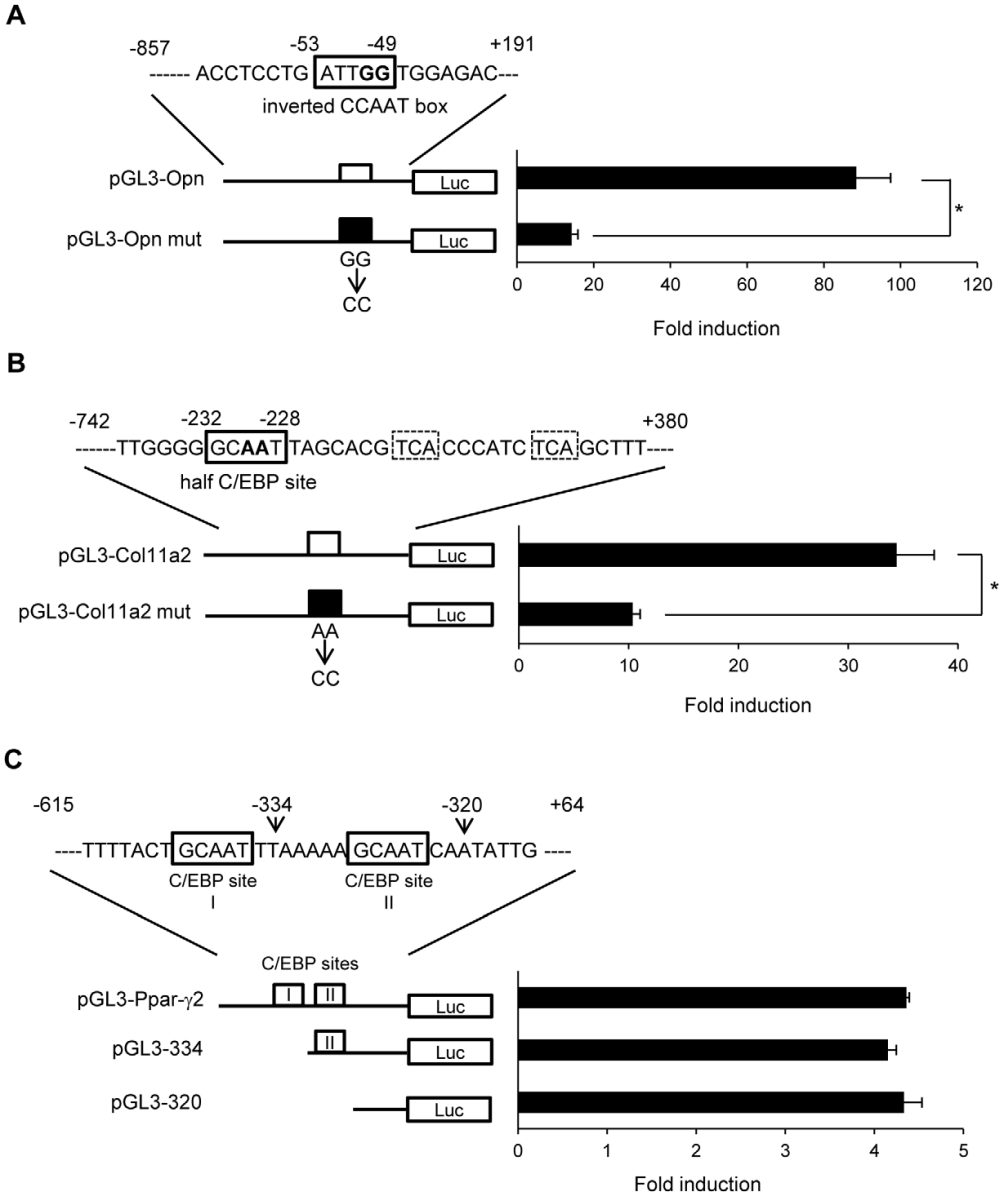
Identification of potential C/EBP-binding sites within Opn and Col11a2 promoters in C3H10T1/2 cells. (**A**) An inverted CCAAT box (boxed) of the pGL3-Opn promoter construct was mutated as designated to produce pGL3-Opn mut. The pGL3-Opn mut exhibited significantly reduced promoter activity. (**B**) A half C/EBP site (boxed) of the pGL3-Col11a2 promoter construct was mutated as designated to produce pGL3-Col11a2 mut. The pGL3-Col11a2 mut exhibited significantly reduced promoter activity. (**C**) The pGL3-Ppar-γ2 promoter construct containing tandem repeats of C/EBP-binding sites (boxed) and its deletion mutants, pGL3-334 (lacking the distal C/EBP binding site I) and pGL3-320 (lacking both C/EBP-binding sites I and II), exhibited comparable activity. The luciferase activities were expressed as fold inductions; each activity relative to that of the promoter-less reporter vector (pGL3 basic). Transfection in duplicate was repeated at least three times, and the results are shown as averages ± SE. Asterisks (*) indicate statistical significance (*p*<0.05) calculated by unpaired t-test.

The LZ dimer forming domain and DNA-binding domain of DDIT3 are highly conserved among members of the C/EBP protein family of transcription factors. To identify the DNA sequence within Opn and Col11a2 promoters that the EWSR1-DDIT3 fusion protein might target in multipotent mesenchymal C3H10T1/2 cells, we inspected each promoter sequence and relevant literature. The Opn promoter contained an inverted CCAAT box located at −53 to −49 from the transcription start site, which mediated transcriptional stimulation by v-Src in NIH3T3 cells (Figure 4A) [34]. Inspection of the Col11a2 promoter sequence revealed a novel candidate C/EBP-binding site consisting of two half sites for CREB or AP-1, 5′-TCA-3′, located at −220 to −218 and −211 to −209, and C/EBP half-site, 5′-GCAAT-3′, located at −232 to −228 from the transcription start site (Figure 4B) [22]. Activation of the mouse alpha 1 chain of the type X collagen (Col10a1) gene by C/EBP β via these two half sites for CREB or AP-1 and the C/EBP half-site was shown in ATDC5 cells [35]. Mutating the inverted CCAAT box in the Opn promoter construct to aaAAT (Opn mut) and the C/EBP half-site in the Col11a2 promoter construct to GCccT (Col11a2 mut) significantly reduced the luciferase activity by 84% and 70%, respectively, when transiently transfected into C3H10T1/2 cells (Figure 4A and 4B). In contrast, Ppar-γ2 promoter activity was not significantly influenced by deleting tandem repeats of C/EBP-binding sites located between −615 to −320, which were shown to mediate glucocorticoid-induced adipocytic differentiation of C3H10T1/2 cells (Figure 4C) [36]. These findings suggest that Opn and Col11a2 promoter activities depended on potential C/EBP-binding sites, which could be targets for EWSR1-DDIT3 in innate mouse multipotent mesenchymal C3H10T1/2 cells. Further experiments using hMSCs showed that mutating the putative C/EBP-binding sites significantly decreased transcriptional activities from Opn, Col11a2, and Ppar-γ2 promoters (Figure S1A–C). These observations, unlike those in C3H10T1/2 cell line, indicated that hMSCs positively utilized the proximal C/EBP-binding site of the tandem repeat within the Ppar-γ2 promoter.

### EWSR1-DDIT3 fusion protein bound potential C/EBP-binding sites within Opn and Col11a2 promoters in vivo

**Figure 5:**
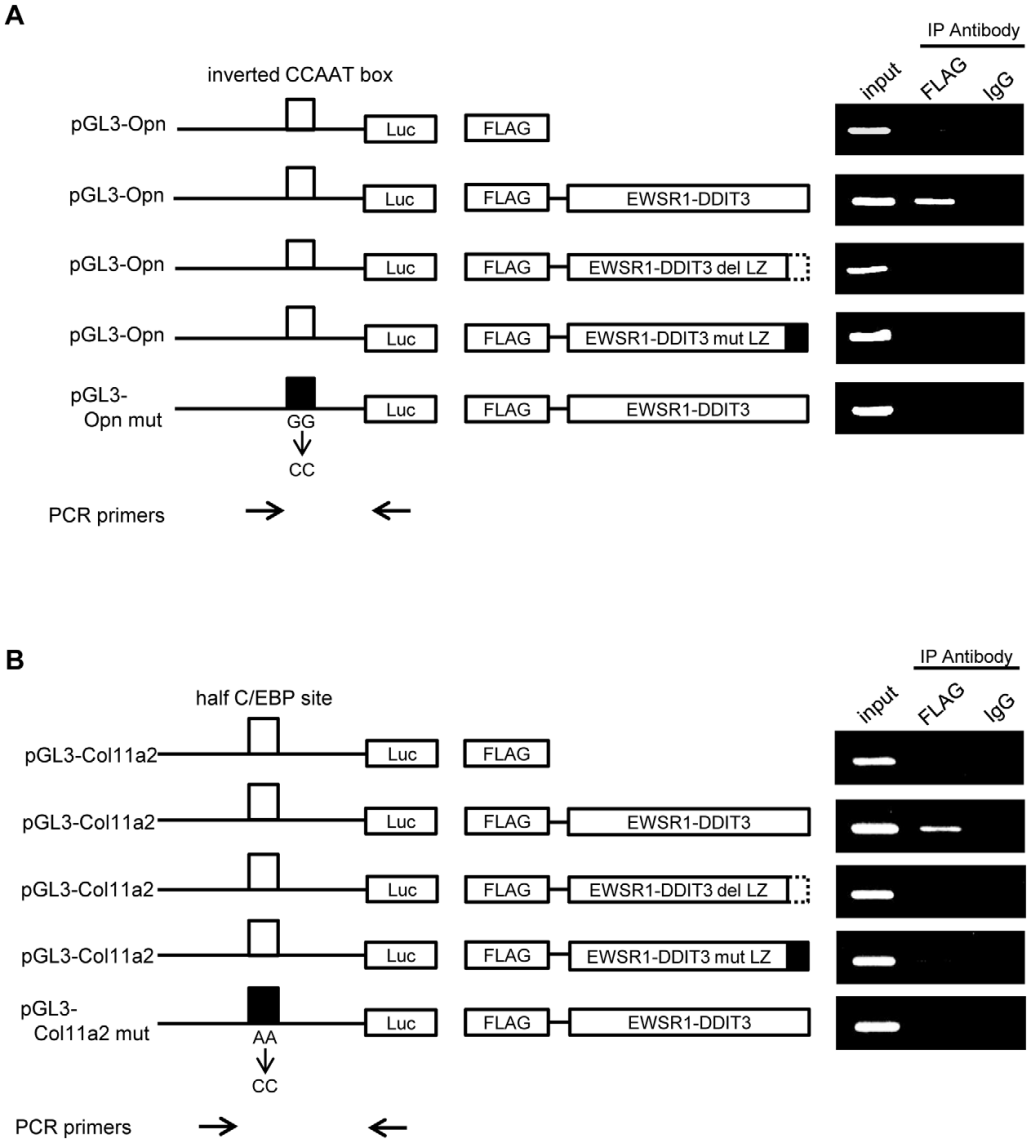
Interaction of EWSR1-DDIT3 and potential C/EBP-binding sites within Opn and Col11a2 promoters *in vivo*. Transient ChIP assays using an antibody against the N-terminal FLAG epitope or normal IgG. C3H10T1/2 cells were transfected with Opn (**A**) or Col11a2 (**B**) luciferase reporter plasmids plus FLAG-tagged expression vectors. Promoter DNA fragments containing potential C/EBP-binding sites (open box) were immunoprecipitated with EWSR1-DDIT3 when wild-type Opn (pGL3-Opn) and Col11a2 (pGL3-Col11a2) promoter constructs were analyzed. Either deleting (EWSR1-DDIT3 del LZ, broken line) or mutating (EWSR1-DDIT3 mut LZ, filled box) the LZ dimer forming domain of EWSR1-DDIT3 or mutating potential C/EBP-binding sites within Opn (inverted CCAAT box, pGL3-Opn mut, filled box) and Col11a2 (half C/EBP site, pGL3-Col11a2 mut, filled box) promoters eliminated the protein–DNA interaction. As indicated by the arrows, the forward PCR primers are promoter sequence-specific primers and locate upstream to the potential C/EBP-binding site (open box), while the reverse PCR primer pGL3 is a plasmid-specific primer.

To explore further the role of EWSR1-DDIT3 in the regulation of Opn and Clo11a2 promoters *in vivo*, we performed transient chromatin immunoprecipitation (ChIP) assays [37] with some modifications. Use of transient ChIP assays that measure the interaction of proteins with plasmid-based sequences allowed the determination of the role of specific local elements in the interaction of EWSR1-DDIT3 with Opn and Col11a2 promoters [38], [39]. C3H10T1/2 cells were transiently transfected with the various reporter constructs described above in permutational combinations with the various FLAG-tagged EWSR1-DDIT3 expression vectors. Promoter sequences associated with FLAG-tagged proteins were immunoprecipitated from the soluble nuclear material containing reporter plasmids, which was cross-linked by formaldehyde and enzymatically sheared to 200–1500 bp fragments, and then analyzed by semiquantitative PCR using primers specific for the reporter construct. Immunoprecipitation performed using normal immunoglobulin (IgG) served as a negative control. Using plasmid-specific primers, we were able to distinguish the reporter constructs from the endogenous gene promoters. As expected from the results of the abovementioned promoter-luciferase reporter assays, FLAG-tagged EWSR1-DDIT3 interacted with wild-type Opn and Col11a2 promoters, while EWSR1-DDIT3 del LZ and EWSR1-DDIT3 mut LZ did not (Figure 5A and 5B). Importantly, mutation of the potential C/EBP site within each construct eliminated the binding of wild-type EWSR1-DDIT3 to the promoter. These results indicated that EWSR1-DDIT3 bound to the potential C/EBP site within Opn and Col11a2 constructs thorough the DNA-binding domain and repressed gene transcription *in vivo*.

### EWSR1-DDIT3 fusion protein affected the binding of endogenous C/EBPβ, but not that of C/EBPα, to C/EBP-binding sites within Opn and Col11a2 promoters

**Figure 6:**
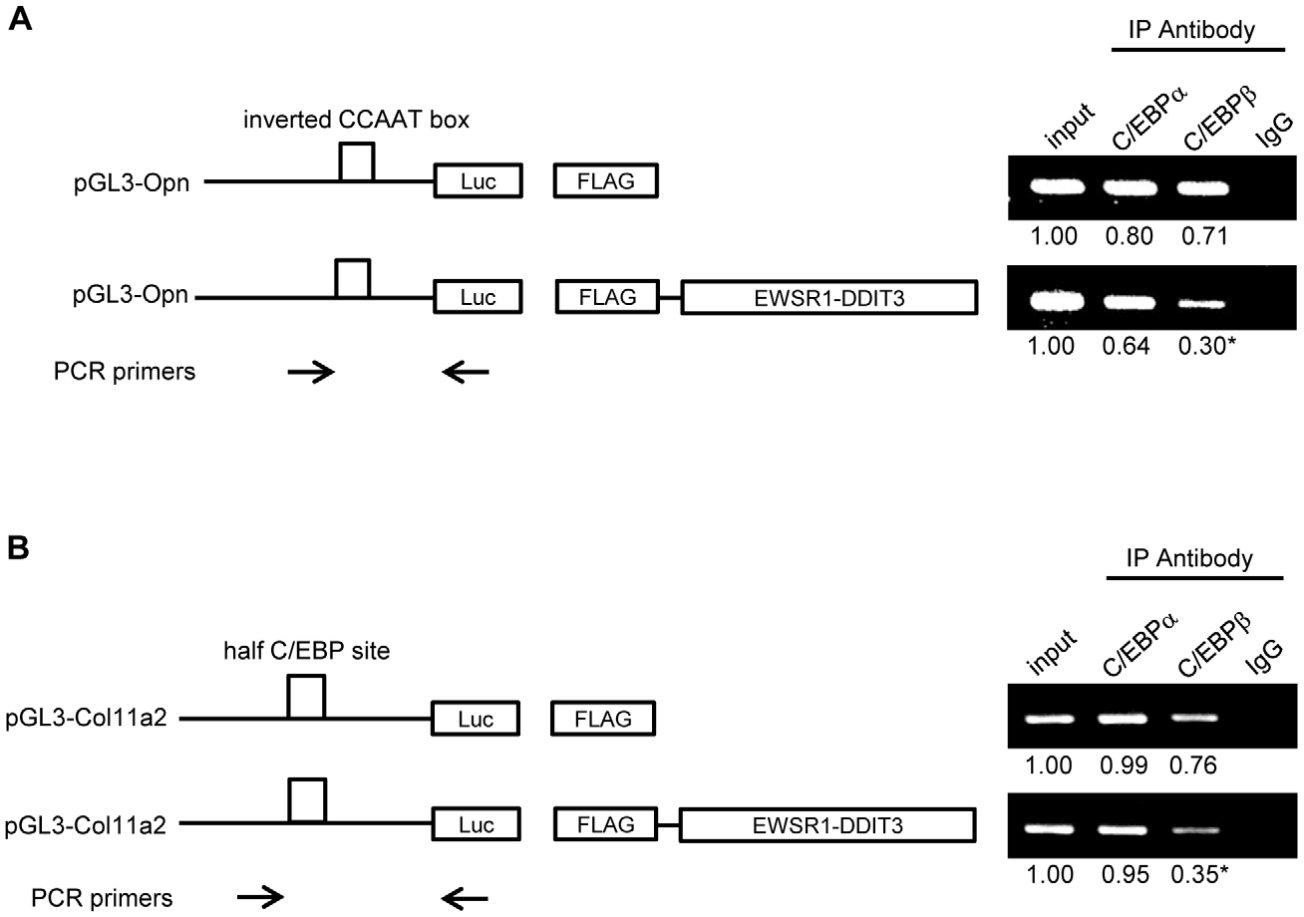
EWSR1-DDIT3 affected recruitment of endogenous C/EBPβ to the C/EBP site within Opn and Col11a2 promoters. Transient ChIP assays using an antibody against C/EBPα, C/EBPβ, or normal IgG. C3H10T1/2 cells were transfected with Opn (**A**) and Col11a2 (**B**) luciferase reporter plasmids plus EWSR1-DDIT3 expression vectors. Promoter DNA fragments containing the C/EBP site (open box) were immunoprecipitated with an antibody against C/EBPα or C/EBPβ. Relative values reflecting protein–DNA interactions were calculated by adjusting corresponding signal intensities to those of input levels. Experiments in duplicate were repeated at least three times, and the results are shown as averages below each band. For C/EBPβ, the relative value of immunoprecipitated Opn and Col11a2 promoter fragments significantly decreased after EWSR1-DDIT3 overexpression from 0.71 to 0.30 and 0.76 to 0.35, respectively. Asterisks (*) indicate statistical significance (*p*<0.05) calculated by unpaired *t*-test, with *p* values of 0.022 for Opn and 0.0025 for Col11a2. As indicated by the arrows, the forward PCR primers are promoter sequence-specific primers and locate upstream to the C/EBP site (open box), while the reverse PCR primer pGL3 is a plasmid-specific primer.

DDIT3 forms heterodimers with a variety of other LZ dimer forming domain-containing transcription factors. The DDIT3-derived fusion proteins also form heterodimers. C/EBPα and C/EBPβ were detectable at protein level in C3H10T1/2 cells, and C/EBPβ was more abundantly expressed than C/EBPα at mRNA level (Figure S2A and S2B). Based on our earlier observations showing the critical role of C/EBP-binding sites within Opn and Col11a2 promoters for gene transcription in innate mouse multipotent mesenchymal C3H10T1/2 cells and *in vivo* binding of EWSR1-DDIT3 to C/EBP-binding sites within Opn and Col11a2 promoters, we speculated that EWSR1-DDIT3 might affect the function of endogenous C/EBP protein family of transcription factors. To address this possibility, we continued the transient ChIP assay using antibodies against C/EBPα and C/EBPβ in cells transfected with the empty CMV promoter-FLAG plasmid or FLAG-tagged EWSR1-DDIT3 expression vector together with Opn and Col11a2 reporter constructs. Immunoprecipitation performed using IgG served as a negative control. As shown in Figure 6A and 6B, in the absence of EWSR1-DDIT3, both C/EBPα and C/EBPβ were able to bind to C/EBP sites containing segments of Opn and Col11a2 promoters. Interestingly, EWSR1-DDIT3 overexpression reduced the amount of promoter fragments immunoprecipitated with an antibody against C/EBPβ, while it had a negligible impact when an antibody against C/EBPα was used (Figure 6A and 6B). Among the abovementioned two major members of the C/EBP family, previous studies have provided evidence that C/EBPβ may promote osteoblastic differentiation of C3H10T1/2 cells via the C/EBP site [40] and that DDIT3 prevents C/EBPβ binding to the C/EBP site by forming heterodimers [41]. The mRNA level for C/EBPα seemed increased and that for C/EBPβ decreased, with no statistical significance (Figure S2B). Collectively, these data could indicate that EWSR1-DDIT3 represses Opn and Col11a2 gene transcription by interfering, at least in part, with endogenous C/EBPβ function.

### Histone deacetylases were involved in EWSR1-DDIT3-mediated repression of Opn and Col11a2 transcriptional activities

**Figure 7:**
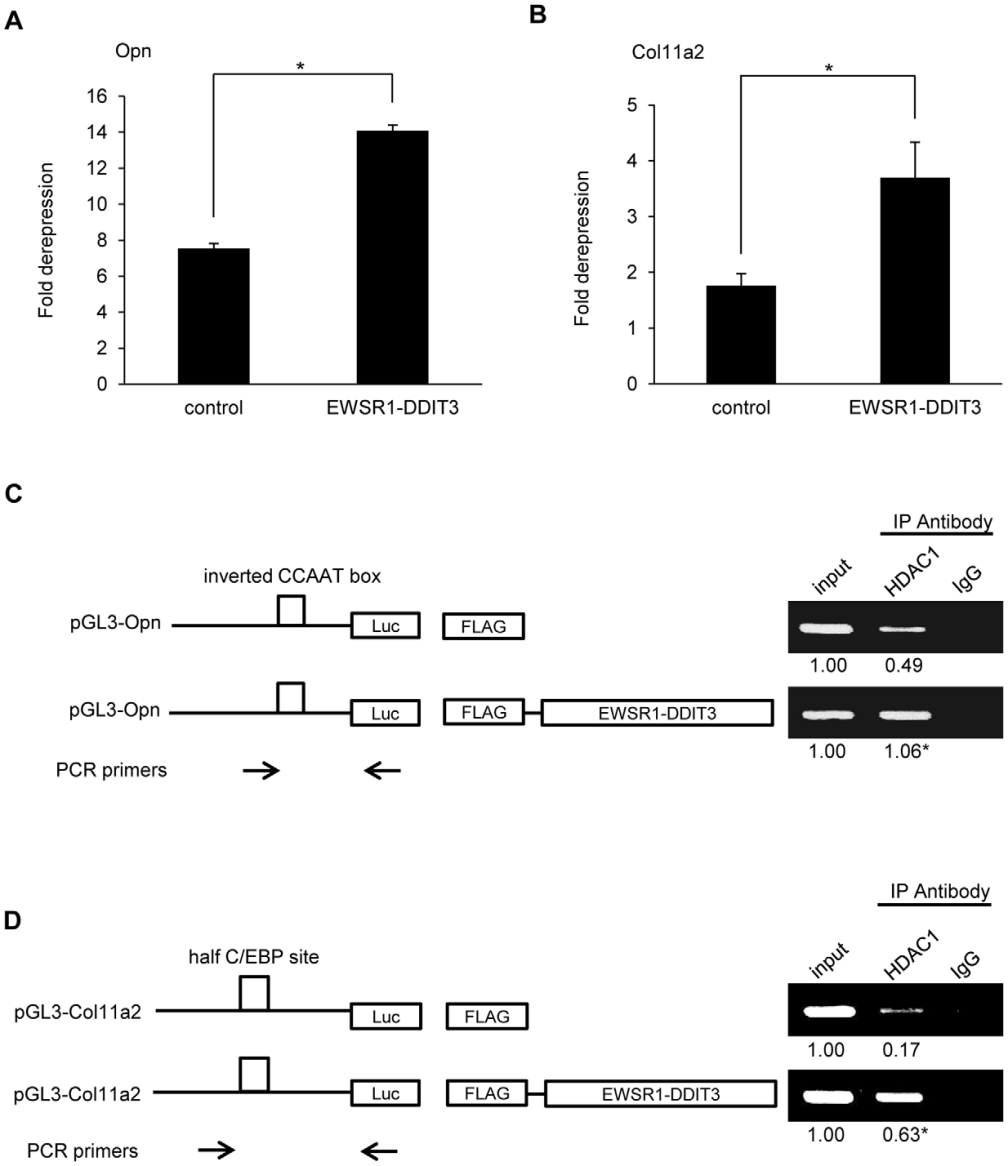
Involvement of histone deacetylases in transcriptional repression of Opn and Col11a2 promoters by EWSR1-DDIT3. (**A** and **B**) Derepression of Opn (**A**) and Col11a2 (**B**) promoter activity by HDAC inhibitor, trichostatin A (TSA). C3H10T1/2 cells in duplicate plates were cotransfected with promoter reporter plasmids plus EWSR1-DDIT3 expression vectors. Cells in one plate were assayed for luciferase activity 24 h after treatment with TSA and compared with the cells from the other plate that were not treated with TSA. Luciferase activities from TSA-treated cells relative to those from TSA-untreated cells are shown as fold derepression. Experiments in duplicate were repeated at least three times, and the results are shown as averages ± SE. Asterisks (*) indicate statistical significance (*p*<0.05) calculated by unpaired *t*-test, with *p* values of 0.0001 for Opn and 0.0277 for Col11a2. (**C** and **D**) Transient ChIP assays using an antibody against HDAC1 or normal IgG. C3H10T1/2 cells were transfected with Opn (**C**) and Col11a2 (**D**) luciferase reporter plasmids plus EWSR1-DDIT3 expression vectors. Promoter DNA fragments containing the C/EBP site (open box) were immunoprecipitated with an antibody against HDAC1. Relative values reflecting protein–DNA interactions were calculated by adjusting corresponding signal intensities to those of input levels. Experiments in duplicate were repeated at least three times, and the results are shown as averages below each band. Relative values of immunoprecipitated Opn and Col11a2 promoter fragments significantly increased after EWSR1-DDIT3 overexpression from 0.49 to 1.06 and 0.17 to 0.63, respectively. Asterisks (*) indicate statistical significance (*p*<0.05) calculated by unpaired *t*-test, with *p* values of 0.0005 for Opn and 0.0021 for Col11a2. As indicated by the arrows, the forward PCR primers are promoter sequence-specific primers and locate upstream to the C/EBP site (open box), while the reverse PCR primer pGL3 is a plasmid-specific primer.

Histone modifications play a critical role in transcriptional control, and among such modifications, lysine acetylation is generally correlated with transcriptional activation [42], [43]. Histone acetylation levels are controlled by histone acetylases and histone deacetylases (HDACs). To further elucidate the mechanisms by which EWSR1-DDIT3 repressed gene transcription, we investigated the influence of pharmacological modulators of gene expression by treating transfected cells with trichostatin A (TSA), a potent inhibitor of HDACs [44], 24 h after transfection. Transfected cells in control plates were not treated with TSA and were used to calculate fold derepression by TSA. The results showed that the ability of EWSR1-DDIT3 to repress Opn and Col11a2 promoter activities was significantly attenuated by TSA-mediated inhibition of HDACs (Figure 7A and 7B). In addition, transient ChIP assays using an anti-HDAC1 antibody revealed enhanced binding of HDAC1 to C/EBP sites containing segments of Opn and Col11a2 promoters in the presence of EWSR1-DDIT3, when cells transfected with the empty CMV promoter-FLAG plasmid or FLAG-tagged EWSR1-DDIT3 expression vectors plus Opn and Col11a2 reporter constructs were analyzed (Figure 7C and 7D). These results implied the potential involvement of HDACs at the gene repression site by EWSR1-DDIT3 *in vivo*. In contrast, the repressive action of anacardic acid (AA, Sigma), a small molecule compound which inhibits histone acetyltransferase (HAT) activity of p300 and PCAF [45], [46], on promoter activities from both promoters was significantly attenuated by overexpressing EWSR1-DDIT3 (Figure S3A and S3B). This observation implies that EWSR1-DDIT3 may interfere with HAT activity of certain promoters at the site of transcription in C3H10T1/2 cells.

### DNA methylation contributed to transcriptional repression of Col11a2 by EWSR1-DDIT3

**Figure 8:**
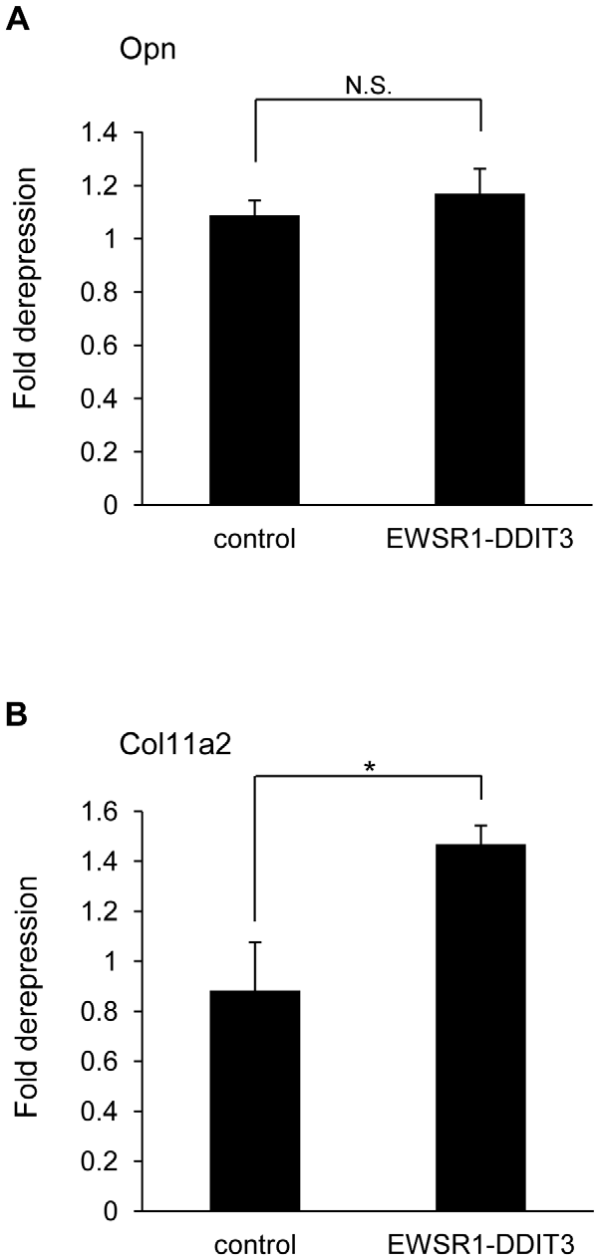
Significant derepression of the Col11a2 promoter, but not the Opn promoter, by 5-Aza-2′-deoxycytidine. (**A** and **B**) Effect of the DNA methylation-resistant cytosine analog 5-Aza-2′-deoxycytidine (AZA) on Opn (**A**) and Col11a2 (**B**) promoter activities. C3H10T1/2 cells in duplicate plates were cotransfected with promoter reporter plasmids plus EWSR1-DDIT3 expression vectors. Cells in one plate were assayed for luciferase activity 24 h after treatment with AZA and compared with the cells from the other plate that were not treated with AZA. Luciferase activities from AZA-treated cells relative to those from AZA-untreated cells are shown as fold derepression. Experiments in duplicate were repeated at least three times, and the results are shown as averages ± SE. An asterisk (*) indicates statistical significance (*p*<0.05) calculated by unpaired *t*-test, with a *p* value of 0.0301 for Col11a2. N.S., not significant.

DNA methylation is another category of epigenetic modification [43] and has been well documented as a mediator of transcriptional repression [47]. DNA methylation and histone deacetylation function synergistically through the formation of transcriptional repressor complexes that silence gene expression by establishing a repressive chromatin environment [48]–[50]. We analyzed the influence of pharmacological modulators of DNA methylation by treating transfected cells with 5-Aza-2′-deoxycytidine (AZA), a methylation-resistant cytosine analog [51], 24 h after transfection. Transfected cells in the control plate were not treated with AZA and were used to calculate fold derepression by AZA. Whereas the ability of EWSR1-DDIT3 to repress the Col11a2 promoter deteriorated significantly in the presence of AZA, its ability to repress the Opn promoter was not profoundly influenced by AZA-mediated inhibition of DNA methylation (Figure 8A and 8B). The differential effect of AZA on Opn and Col11a2 promoters might result from differences in their structural class [52], i.e., the Opn promoter contains a TATA-like sequence (TTTAAA) at −27 to −22 from the transcription start site [53], while the Col11a2 promoter does not have a proper TATA box [22] but rather contains several SP1-binding sites [54], typical of such TATA-less class promoters [55]. These data indicated a potential involvement of DNA methylation in repression of TATA-less and high-CpG Col11a2 promoter by EWSR1-DDIT3. On the other hand, adding temozolomide (TMZ, Sigma), a DNA methylating chemotherapeutic drug [56], [57], did not apparently influence the activity from both promoters in 10T1/2 cells (Figure S4A and S4B).

### EWSR1-DDIT3 influenced levels of acetylation or trimethylation at lysine 9 of histone 3 around Opn and Col11a2 promoters

**Figure 9:**
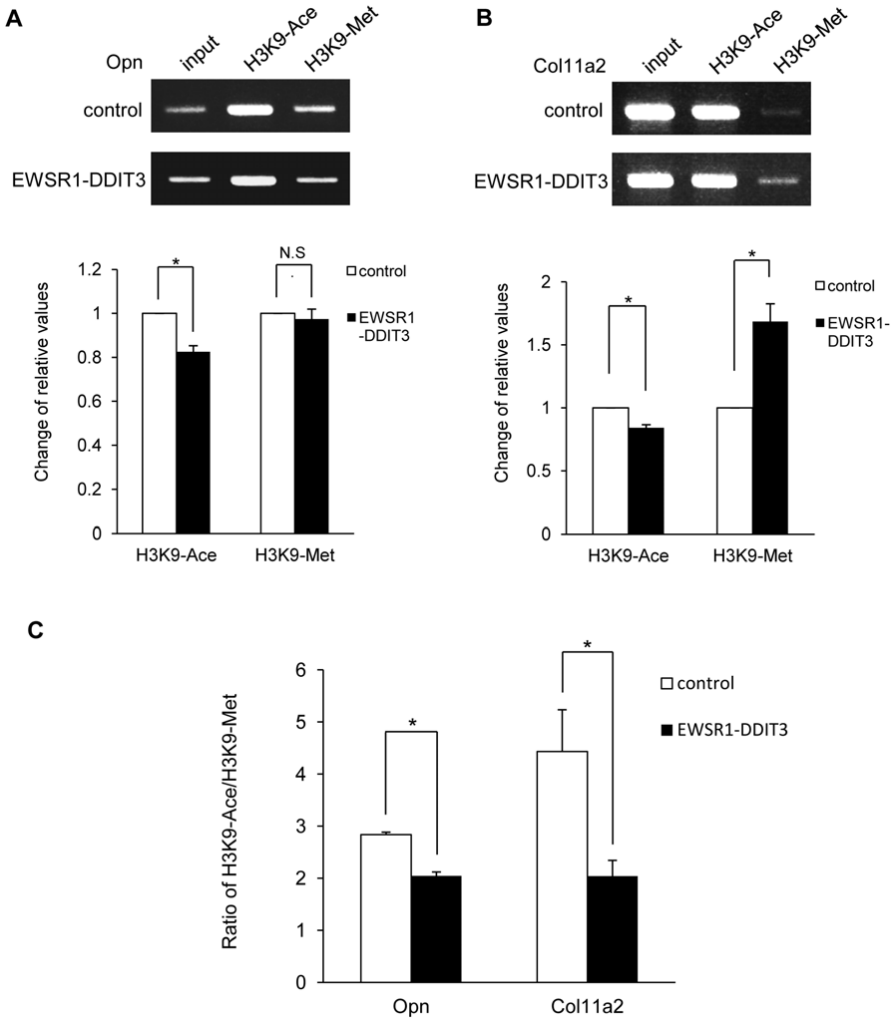
EWSR1-DDIT3 influenced the levels of acetylation or trimethylation at H3K9 around Opn and Col11a2 promoters. Transient ChIP assays using an antibody against acetylated H3K9 or trimethylated H3K9. C3H10T1/2 cells were transfected with Opn (**A**) and Col11a2 (**B**) luciferase reporter plasmids plus EWSR1-DDIT3 expression vectors. Promoter DNA fragments around C/EBP site were immunoprecipitated with an antibody against acetylated H3K9 (H3K9-Ace) or trimethylated H3K9 (H3K9-Met) (top panels). Relative values reflecting protein–DNA interactions were calculated by adjusting corresponding signal intensities to those of input levels. Experiments in duplicate were repeated at least three times, and EWSR1-DDIT3-mediated changes in relative values are shown as averages ± SE (bottom panels). (**C**) Ratio of H3K9-Ace versus H3K9-Met around Opn and Col11a2 promoter constructs. Error bars indicate SE. Asterisks (*) indicate statistical significance (*p*<0.05) calculated by unpaired *t*-test. N.S., not significant.

Histone deacetylation, DNA methylation, and methylation at lysine 9 of histone 3 (H3K9) are the best-characterized covalent modifications associated with a repressed chromatin state [58], [59]. We assessed the levels of acetylation or trimethylation at H3K9 around Opn and Col11a2 promoters by transient ChIP assays in cells transfected with the empty CMV promoter-FLAG plasmid or FLAG-tagged EWSR1-DDIT3 expression vectors together with Opn and Col11a2 reporter constructs. Our data demonstrated that EWSR1-DDIT3 overexpression decreased the amount of Opn and Col11a2 promoter fragments immunoprecipitated with an antibody against acetylated H3K9 (Figure 9A and 9B). When an antibody against trimethylated H3K9 was used, relative values of protein–DNA interaction for Col11a2 were increased by EWSR1-DDIT3, while those for Opn did not change (Figure 9A and 9B). The ratio of acetylated H3K9 to trimethylated H3K9 around Opn and Col11a2 promoter segments was significantly decreased by EWSR1-DDIT3 (Figure 9C), which reflected epigenetic silencing of gene transcription [60]–[62].

## Discussion

It is becoming increasingly evident that many transforming genetic alterations responsible for sarcoma development are found in multipotent mesenchymal cells [2]–[5]. The induction of several types of sarcomas, such as EWSR1, MLS, and alveolar rhabdomyosarcoma, by overexpression of specific oncogenic fusion proteins in multipotent mesenchymal cells has been documented [63]–[66]. However, the exact molecular mechanisms by which these fusion proteins exert oncogenic transformation through phenotypic selection of the uncommitted target cells remain uncertain.

**Figure 10:**
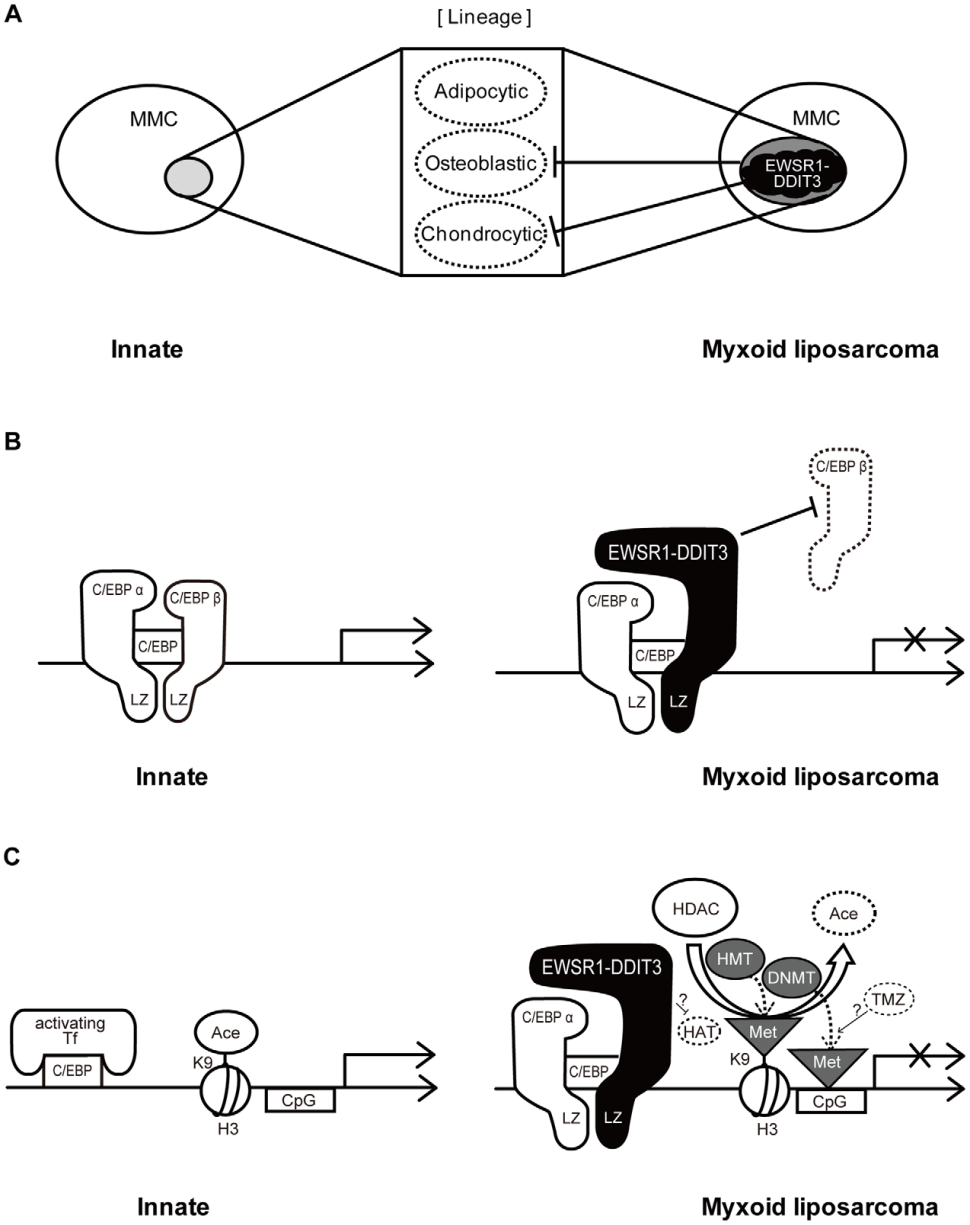
Hypothetical diagram showing mechanisms by which EWSR1-DDIT3 exerts selective transcriptional repression in multipotent mesenchymal cells. (**A**) Direct lineage reprogramming of multipotent mesenchymal cells (MMC) by EWSR1-DDIT3. (**B**) Genetic action of EWSR1-DDIT3, which binds to the functional C/EBP site within target promoters through interaction of its DNA-binding domain and interferes with endogenous C/EBPβ function. (**C**) Epigenetic action introduced by EWSR1-DDIT3, enhancing histone deacetylation, DNA methylation, and histone 3 (H3) lysine 9 (K9) trimethylation at the transcriptional repression site. Tf, transcription factor; Ace, acetylation; CpG, cytosine–guanine dinucleotide; HAT, histone acetyltransferase; HDAC, histone deacetylases; HMT, histone methyltransferases; DNMT, DNA methyltransferases; Met, methylation; TMZ, temozolomide.

In this study, we showed that the EWSR1-DDIT3 myxoid liposarcoma fusion protein, but not its wild-type counterparts EWSR1 and DDIT3, selectively repressed the transcriptional activity of cell lineage-specific marker genes in multipotent mesenchymal C3H10T1/2 cells. Specifically, the osteoblastic marker Opn promoter and chondrocytic marker Col11a2 promoter were repressed, while the adipocytic marker Ppar-γ2 promoter was not affected (Figure 2). Cell phenotype is determined by gene expression patterns, and the ability of a cell to change its phenotype outside its lineage is achieved by the activity of transcriptional regulators capable of reprogramming gene networks [67]. Therefore, the above observation, selective transcriptional repression by EWSR1-DDIT3, could partly reflect the ability of EWSR1-DDIT3 to directly reprogram lineages of multipotent mesenchymal cells (Figure 10A).

Previous studies have established the oncogenic potential of TLS-DDIT3, another MLS-associated fusion protein [17], [68]. Our detailed inspection of supporting information tables from a recent microarray-based analysis of TLS-DDIT3-transformed adipose-derived mesenchymal stem cells revealed downregulation of Opn and Col11a2 mRNA levels and preservation of Ppar-γ gene expression [18]. TLS belongs to the TET family of RNA-binding proteins that consists of TLS, EWSR1, and TAF15 [69]. It is possible to assume that EWSR1-DDIT3 functions in a manner similar to TLS-DDIT3, because the two have the conserved SYQG-rich amino terminal domain from EWSR1 or TLS, in addition to share the same DNA-binding domain from DDIT3. If so, our observations are consistent with these data.

This study also elucidated the possible molecular mechanisms underlying the above discussed selective transcriptional repression. First, the EWSR1-DDIT3 fusion protein (Figure 10B) might bind to the functional C/EBP site within Opn and Col11a2 promoters through interaction of its DNA-binding domain and interfere with endogenous C/EBPβ function (Figures 3, 4, 5 and 6). Of note, previous observations showed that the analogous TLS-DDIT3 fusion protein prevented adipocytic differentiation by directly interfering with C/EBPβ function [70] and induced C/EBPβ-mediated interleukin 6 expression through heterodimerization [71]. Second, EWSR1-DDIT3 (Figure 10C) might act in an epigenetic manner to enhance histone deacetylation, DNA methylation, and histone H3K9 trimethylation at the transcriptional repression site (Figures 7, 8 and 9). The epigenetic status has been shown to influence the differentiation propensity of transcription factor-based, directly reprogrammed inducible multipotent stem cells [72]. Thus, we believe that EWSR1-DDIT3-mediated transcriptional regulation may modulate the target cell lineage through target gene-specific genetic and epigenetic conversions.

The reason why Ppar-γ2 promoter activity was not affected by the EWSR1-DDIT3 fusion protein in mouse multipotent mesenchymal C3H10T1/2 cells (Figure 2D) is not clear. However, the observation that this activity seemed independent of C/EBP sites in innate C3H10T1/2 cells (Figure 4C) suggests that EWSR1-DDIT3 might selectively target C/EBP site-dependent transcription. The related TLS-DDIT3 fusion protein has been shown to repress Ppar-γ2 promoter activity in U2OS human bone sarcoma-derived cells [73] but not affect Ppar-γ gene expression in mouse bone marrow-derived mesenchymal progenitor cells [65]. These observations indicate that the molecular action of MLS-associated fusion proteins may manifest in a cell type- and/or species-dependent manner.

In conclusion, this study provides evidence that helps elucidate the molecular mechanisms that underlie the contribution of the EWSR1-DDIT3 fusion protein to the phenotypic selection of targeted multipotent mesenchymal cells during MLS development. These findings are fundamental to achieving a better understanding of possible direct lineage reprogramming in oncogenic sarcoma transformation mediated by fusion proteins.

## Materials and Methods

### Cell culture and RT-PCR

C3H10T1/2 cells (American Type Culture Collection (ATCC), USA) were cultured in Dulbecco's modified Eagle's medium (DMEM) with 10% fetal bovine serum (FBS), penicillin (100 U/ml), and streptomycin (100 µg/ml) under 5% CO_2_ at 37°C. Total RNA was extracted using the SV Total RNA Isolation System (Promega, Madison, WI, USA), and 1.5 µg of total RNA was converted to cDNA using the GeneAmp® Gold RNA PCR Core Kit (Applied Biosystems, Foster City, CA, USA) in accordance with the manufacturer's instructions. The cDNA was advanced to PCR amplification using Platinum® Blue PCR SuperMix (Invitrogen Life Technologies Corp., Carlsbad, CA, USA) and primer sets specific for Opn, Col11a2, Ppar-γ, and β-actin genes. Primer sequences are summarized in Table 1. Each reaction mixture contained 1 µl of cDNA, 20 µl of Platinum® Blue PCR SuperMix, and 10 pmol of each primer. Denaturation for 2 min at 95°C was followed by 30 cycles of 30 s at 95°C, 30 s at 60°C, and 30 s at 72°C, and a final extension of 7 min at 72°C. Aliquots of the PCR products were electrophoresed on 2% agarose gels, visualized by ethidium bromide staining, and directly sequenced using the ABI PRISM® BigDye® Terminator v3.1 Cycle Sequencing Kit (Applied Biosystems) to confirm correct amplification in each reaction.

**Table 1:** Primer Sets Used for RT-PCR.

Gene	Direction	Sequence	GenBank Accession No.
Osteopontin	Forward	5′-ttacagcctgcacccagatcc-3′	NM_009263.2
	Reverse	5′-cgtccatgtggtcatggctttc-3′	
Col11a2	Forward	5′-aggagccccagaagcagt-3′	NM_009926.1
	Reverse	5′-tggcaagggactggactc-3′	
Ppar-γ2	Forward	5′-aggccgagaaggagaagctgttg-3′	NM_011146.3
	Reverse	5′-tggccacctctttgctctgctc-3	
β-actin	Forward	5′-catccgtaaagacctctatgccaac-3′	NM_007393.3
	Reverse	5′-atggagccaccgatccaca-3′	

### Plasmid constructs

The pGL3-Opn promoter construct was a gift from Dr. Piia Aarnisalo, Helsinki, Finland [74]. The pGL3-Col11a2 promoter construct has been described previously [54], [75], [76]. The pGL3-Ppar-γ2 promoter construct containing tandem repeats of C/EBP-binding sites and its deletion mutants, pGL3-334 (lacking the distal C/EBP-binding site) and pGL3-320 (lacking both C/EBP-binding sites), were gifts from Dr. Xu Cao, Birmingham, AL, USA [36]. Variants of pGL3-Opn (pGL3-Opn mut) and pGL3-Col11a2 (pGL3-Col11a2 mut) were created by mutating potential C/EBP-binding sites using the Gene Editor™ Site-Directed Mutagenesis kit (Promega), and successful mutagenesis was confirmed by DNA sequencing. Primer sequences for mutagenesis will be forwarded on request.

**Figure 11:**
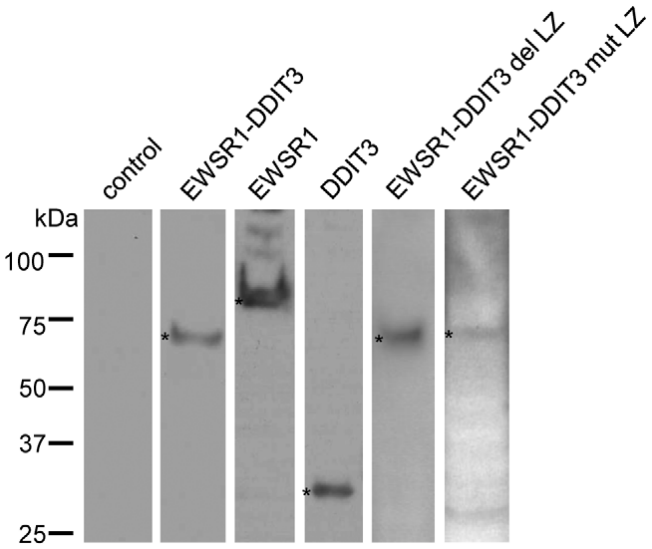
Protein expression by each FLAG-tagged expression vector. Western blotting using an antibody against the N-terminal FLAG epitope tag. Each protein band is indicated by an asterisk (*).

Full-length EWSR1-DDIT3 cDNA, in which exon 7 of EWSR1 was in-frame fused to exon 2 of DDIT3 with a serine (AGT) to methionine (ATG) transition at the junction, was amplified by RT-PCR from cDNA of MLS samples collected from the left thigh of a 19-year-old female [16] using the forward primer EWSR1 exon 1F 5′-aatggcgtccacggattacagtacc-3′ and reverse primer DDIT3 exon 4R 5′-tcatgcttaatacagattcaccattcg-3′. The products were cloned into a pCR®2.1-TOPO® vector using the TOPO TA Cloning® Kit (Invitrogen Life Technologies Corp.), and the correct sequences were confirmed by DNA sequencing. The EcoRI fragment containing full-length EWSR1-DDIT3 cDNA from correct clones was in-frame inserted into the EcoRI site of the pFLAG-CMV4 mammalian expression vector (Sigma) to produce pFLG-CMV4 EWSR1-DDIT3. Full-length EWSR1 cDNA was amplified from human placenta using the forward primer EWSR1 exon 1F and reverse primer EWSR1 exon 17R 5′-ctagtagggccgatct ctgcgctc-3′. Full-length DDIT3 cDNA was amplified from the abovementioned MLS sample using the forward primer DDIT3 exon 2F 5 ′-atgttcaagaaggaagtgtatcttc-3′ and reverse primer DDIT3 exon 4R. The cDNAs were similarly processed to produce pFLAG-CMV4 EWSR1 and pFLAG-CMV4 DDIT3 expression vectors. The resultant proteins were FLAG epitope tagged at the N termini. Two forms of mutant EWSR1-DDIT3 expression vectors were generated by mutating pFLG-CMV4 EWSR1-DDIT3 using the Gene Editor™ Site-Directed Mutagenesis kit (Promega), and successful mutagenesis was confirmed by DNA sequencing. pFLAG-CMV4 EWSR1-DDIT3 del LZ contained a stop codon just 5′ to the codon for the first leucine residue of the dimer forming LZ domain, so that the entire LZ domain, composed of 38 C-terminal amino acid residues, was deleted. Regarding pFLAG-CMV4 EWSR1-DDIT3 mut LZ, all five codons for leucine residues in the LZ domain were changed to codons for glycine residues. Each correct FLAG-tagged protein expression after transient transfection was confirmed by Western blotting using monoclonal anti-FLAG® M2 (Clone M2) antibody (F3165; Sigma) (Figure 11).

### Transfection and luciferase assay

Transient transfection experiments were performed in C3H10T1/2 cells seeded on 6-well plates using X-tremeGENE 9 reagent (Roche Molecular Biochemicals, Indianapolis, IN, USA) with 875 ng pGL3 reporter plasmid, 125 ng pFLAG-CMV4 expression vector, and 30 ng pRL-TK Renilla internal control plasmid (Promega), according to the manufacturer's instructions. For luciferase assays, the cells were harvested 48 h after transfection and luciferase activity was monitored using the Dual Luciferase® Reporter Assay System (Promega) on a TD-20/20 Luminometer (Turner Designs, Sunnyvale, CA, USA). Transfection in duplicate was repeated at least three times, and the luciferase activity was normalized to internal controls. The results are shown as average ± standard errors. For inhibition of HDACs or DNA methytransferases, transfected cells were treated with 100 ng/ml TSA (Sigma) or 5 µM AZA (Sigma), respectively, 24 h before the luciferase assay and compared with transfected cells without TSA or AZA treatment.

### Western blotting

Cells transfected with each pFLAG-CMV4 expression vector in a 10-cm dish were lysed with 1 ml of buffer A (10 mM Tris, pH 7.5, 100 mM NaCl, 2.5 mM MgCl_2_, 0.5% Triton X-100, and 10 mM dithiothreitol (DTT)) supplemented with protease inhibitor cocktail and phosphatase inhibitor cocktail (Sigma). Twenty microliters of 2× SDS sample buffer and 4 µl of DTT were added to 20 µl of the cell lysate. The sample mixtures were denatured for 5 min at 95°C, separated by SDS-PAGE using a 5–15% gradient gel (Bio-Rad Laboratories, Hercules, CA, USA), transblotted onto a polyvinylidene fluoride (PVDF) membrane (Bio-Rad Laboratories), and subjected to Western blotting using monoclonal anti-FLAG® M2 (Clone M2) antibody (F3165; Sigma). Protein bands were visualized on an X-ray film using the ECL Western blotting detection system (GE Healthcare, Buckinghamshire, UK) (Figure 11).

### Transient ChIP assay

This is a well-accepted technique developed to study *in vivo* binding of transcription factors to specific sites on promoters of reporter constructs [37]–[39], [77], [78]. The ChIP-IT™ Express Enzymatic kit (Active Motif, Carlsbad, CA, USA) was used according to the manufacturer's instructions with minor modifications. In brief, C3H10T1/2 cells plated on three 10-cm dishes were transiently transfected with 5 µg pGL3 reporter plasmid together with 5 µg pFLAG-CMV4 expression vector per dish. The cells were maintained for 48 h and cross-linked with 1% formaldehyde for 5 min at room temperature. Soluble nuclear material containing reporter plasmids was collected, enzymatically sheared for 10 min at 37°C, and then immunoprecipitated with 3 µg antibody against N-terminal FLAG epitope tags (F3165; Sigma), C/EBPα (14AA, sc-61X; Santa Cruz Biotechnology Inc., Santa Cruz, CA, USA), C/EBPβ (C-19, sc-150X; Santa Cruz Biotechnology Inc.), HDAC1 (C-19, sc-6298X; Santa Cruz Biotechnology Inc.), acetyl-Histone H3 (Lys9) (07-352; Millipore, Billerica, MA, USA), trimethyl-Histone H3 (Lys9) (07-442; Millipore), or normal IgG (Santa Cruz Biotechnology Inc.). After cross-linking was reversed, associated plasmid DNA was collected, cleaned, and subjected to semiquantitative PCR using primer pairs flanking C/EBP-binding sites within Opn and Col11a2 promoters. The PCR products were electrophoresed on agarose gels and visualized by ethidium bromide staining. Signal intensities were measured using ImageJ software (NIH, Bethesda, MD, USA). Relative values reflecting protein–DNA interactions were calculated by adjusting corresponding signal intensities to those of input levels. PCR primers specific for pGL3-Opn and pGL3-Col11a2 reporters were developed and used to detect associated reporter plasmids in each immunoprecipitation. Primer sequences included the following: Opn F3 (forward; located at −134 to −112 from the transcriptional start site), 5′-ccacaaaaccagaggaggaagtg-3′; Col11a2 F2 (forward; located at −269 to −250 from the transcriptional start site), 5 ′-ttctgcttcacctagtccag-3 ′; and GL primer 2 (reverse; located within the pGL3 plasmid backbone), 5′-ctttatgttttggcgtcttcca-3′.

### Immunoprecipitation and Western blotting

C3H10T1/2 cells transfected with the empty CMV promoter-FLAG control plasmid or FLAG-tagged EWSR1-DDIT3 expression plasmid in a 10-cm dish were lysed with 1 ml of IP Lysis Buffer (PIRCE, Rockford, IL, USA) supplemented with protease inhibitor cocktail and phosphatase inhibitor cocktail (Sigma). Immunoprecipitation of C/EBPα and C/EBPβ was performed using Dynabeads Protein A (Invitrogen Life Technologies Corp.) according to manufacturer's protocol. In brief, to 50 µl of Dynabeads Protein A, 20 µl of rabbit polyclonal anti-C/EBPα antibody (14AA, sc-61; Santa Cruz Biotechnology Inc., Santa Cruz, CA, USA) or anti-C/EBPβ antibody (C-19, sc-150; Santa Cruz Biotechnology Inc.) were added and incubated for 1 h, and 500 µg protein of cell lysate added to the Dynabeads Protein A conjugated antibody for overnight on a rotating device at 4°C. Immunoprecipitated proteins were denatured for 5 min at 95°C, separated by SDS-PAGE using a 5–15% gradient gel (Bio-Rad Laboratories, Hercules, CA, USA), transblotted onto a polyvinylidene fluoride (PVDF) membrane (Bio-Rad), and subjected to Western blotting using anti-C/EBPα antibody or anti-C/EBPβ antibody (Santa Cruz). Protein bands were visualized on X-ray film using the ECL Plus Western blotting detection system (GE Healthcare, Buckinghamshire, UK).

### Real-time quantitative PCR assay

After transfection of the empty CMV promoter-FLAG control plasmid or FLAG-tagged EWSR1-DDIT3 expression vector into C3H10T1/2 cells, total RNA was extracted using the SV Total RNA Isolation System (Promega, Madison, WI, USA), and 1 µg of total RNA was converted to cDNA using the GeneAmp® Gold RNA PCR Core Kit (Applied Biosystems, Foster City, CA, USA) with random hexamers as a primer in accordance with the manufacturer's instructions. For quantitative analysis of the expression levels of C/EBPα mRNA and C/EBPβ mRNA, real-time quantitative PCR (qPCR) was performed on a Real-Time PCR system (Mx3000P, Stratagene Japan K.K., Tokyo, Japan) using SYBER Premix Ex Taq™ (TaKaRa Bio, Inc. Shiga, Japan). Real-time qPCR was performed using the specific primers and levels of β-actin transcripts were used to normalize C/EBPα and C/EBPβ expression levels. Primer sequences were as follows: C/EBPα 5′-tgaacaagaacagcaacgag-3′ and 5′-tcactggtcacctccagcac-3′; C/EBPβ, 5′-gcgcgagcgcaacaacatcg-3′ and 5′-tgcttgaacaagttccgcag-3′; β-actin, 5′-tgttaccaactgggacgaca-3′ and 5′-ggggtgttgaaggtctcaaa-3′.

### Statistics

Each sample was analyzed in duplicate, and experiments were repeated at least three times. In all figures, data were shown as average ± standard errors (SE). All statistical analyses were performed using Microsoft Office Excel (Microsoft Corp., Redmond, WA, USA). ANOVA followed by Tukey–Kramer post-hoc test or unpaired *t*-test was used to determine statistical significance (*p* values less than 0.05 were considered significant), where applicable.

## Supporting Information

Figure S1 **Identification of potential C/EBP-binding sites within Opn, Col11a2, and Ppar-γ2 promoters in hMSCs.** The pGL3-Opn mut (**A**) and pGL3-Col11a2 mut (**B**) showed significantly reduced promoter activity compared with wild-type promoter constructs. (**C**) The pGL3-Ppar-γ2 promoter construct containing tandem repeat of C/EBP-binding sites and its deletion mutant, pGL3-334 (lacking the distal C/EBP binding site (I) in Figure 4C) exhibited comparable activity. The pGL3-320 (lacking the both C/EBP-binding sites) showed significantly reduced promoter activity. The luciferase activities were expressed as fold inductions; each activity relative to that of the promoter-less reporter vector (pGL3 basic). Transfection in duplicate was repeated at least three times, and the results are shown as averages ± SE. Asterisks (*) indicate statistical significance (*p*<0.05) calculated by unpaired t-test on Opn (*p* = 0.0044) or Col11a2 (*p* = 0.0075) promoter activity and ANOVA on Ppar-γ2 promoter activity following Tukey–Kramer post-hoc test (N.S., not significant, *p<0.05).
(TIF)

Figure S2 **MV4 control or EWSR1-DDIT3.** (**A**) The lysate from C3H10T1/2 cells transfected with pFLAG-CMV4 control or EWSR1-DDIT3 was immunoprecipitated (IP) with an anti-C/EBPα antibody or an anti-C/EBPβ antibody. IP samples were electrophoresed and blotted with an anti-C/EBPα antibody or an anti-C/EBPβ antibody. Each protein band is indicated by an asterisk (*). (**B**) Real-time quantitative PCR assay for the endogenous mRNA levels of C/EBPα and C/EBPβ in C3H10T1/2 cells transfected with pFLAG-CMV4 control or EWSR1-DDIT3. Each mRNA expression level was normalized to that of β-actin. Similar results were obtained in three independent experiments.
(TIF)

Figure S3 **Repression of Opn or Col11a2 promoter activity by anacardic acid (AA), a small molecule compound which inhibits histone acetyltransferase (HAT) activity of p300 and PCAF, was significantly attenuated by overexpressing EWSR1-DDIT3.** Effect of AA on Opn (**A**) and Col11a2 (**B**) promoter activities. C3H10T1/2 cells in duplicate plates were cotransfected with each promoter reporter construct plus EWSR1-DDIT3 expression vector. Cells in one plate were assayed for luciferase activity 24 h after treatment with AA (30 µM) and compared with the cells from the other plate that were not treated with AA. Luciferase activities from AA-treated cells relative to those from AA-untreated cells are shown as fold repression. Experiments in duplicate were repeated at least three times, and the results are shown as averages ± SE. An asterisk (*) indicates statistical significance (*p*<0.05) calculated by unpaired *t*-test, with *p* values of 0.0336 for Opn and 0.0401 for Col11a2.
(TIF)

Figure S4 **Temozolomide (TMZ), a DNA methylating chemotherapeutic drug, did not significantly influenced on the Opn and Col11a2 promoter activities.** Effect of TMZ on Opn (**A**) and Col11a2 (**B**) promoter activities. C3H10T1/2 cells in duplicate plates were cotransfected with each promoter reporter constructs plus EWSR1-DDIT3 expression vector. Cells in one plate were assayed for luciferase activity 44 h after treatment with TMZ (50 µM) and compared with the cells from the other plate that were not treated with TMZ. Luciferase activities from TMZ-treated cells relative to those from TMZ-untreated cells are shown as fold repression. Experiments in duplicate were repeated at least three times, and the results are shown as averages ± SE. N.S., not significant.
(TIF)
