# Supplementary figures and images for: Myxoid Liposarcoma-Associated EWSR1-DDIT3 Selectively Represses Osteoblastic and Chondrocytic Transcription in Multipotent Mesenchymal Cells
Source: PLoS One. 2012 May 3;7(5):e36682. doi: 10.1371/journal.pone.0036682 (PMC3343026; doi:10.1371/journal.pone.0036682)

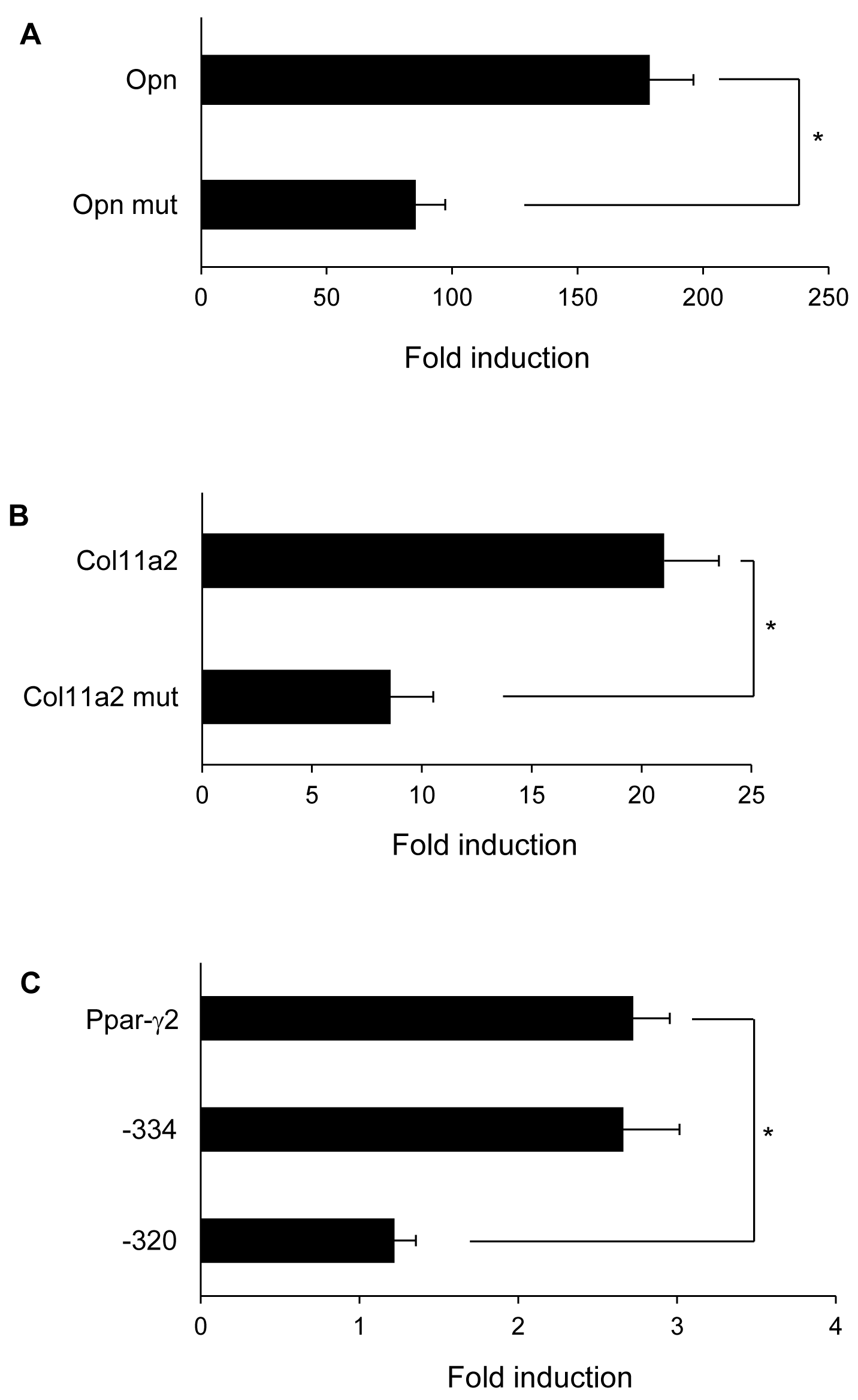

Supplement: Figure S1 — Identification of potential C/EBP-binding sites within Opn, Col11a2, and Ppar-γ2 promoters in hMSCs. The pGL3-Opn mut (A) and pGL3-Col11a2 mut (B) showed significantly reduced promoter activity compared with wild-type promoter constructs. (C) The pGL3-Ppar-γ2 promoter construct containing tandem repeat of C/EBP-binding sites and its deletion mutant, pGL3-334 (lacking the distal C/EBP binding site (I) in Figure 4C) exhibited comparable activity. The pGL3-320 (lacking the both C/EBP-binding sites) showed significantly reduced promoter activity. The luciferase activities were expressed as fold inductions; each activity relative to that of the promoter-less reporter vector (pGL3 basic). Transfection in duplicate was repeated at least three times, and the results are shown as averages ± SE. Asterisks (*) indicate statistical significance (p<0.05) calculated by unpaired t-test on Opn (p = 0.0044) or Col11a2 (p = 0.0075) promoter activity and ANOVA on Ppar-γ2 promoter activity following Tukey–Kramer post-hoc test (N.S., not significant, *p<0.05). (TIF) [file pone.0036682.s001.tif]

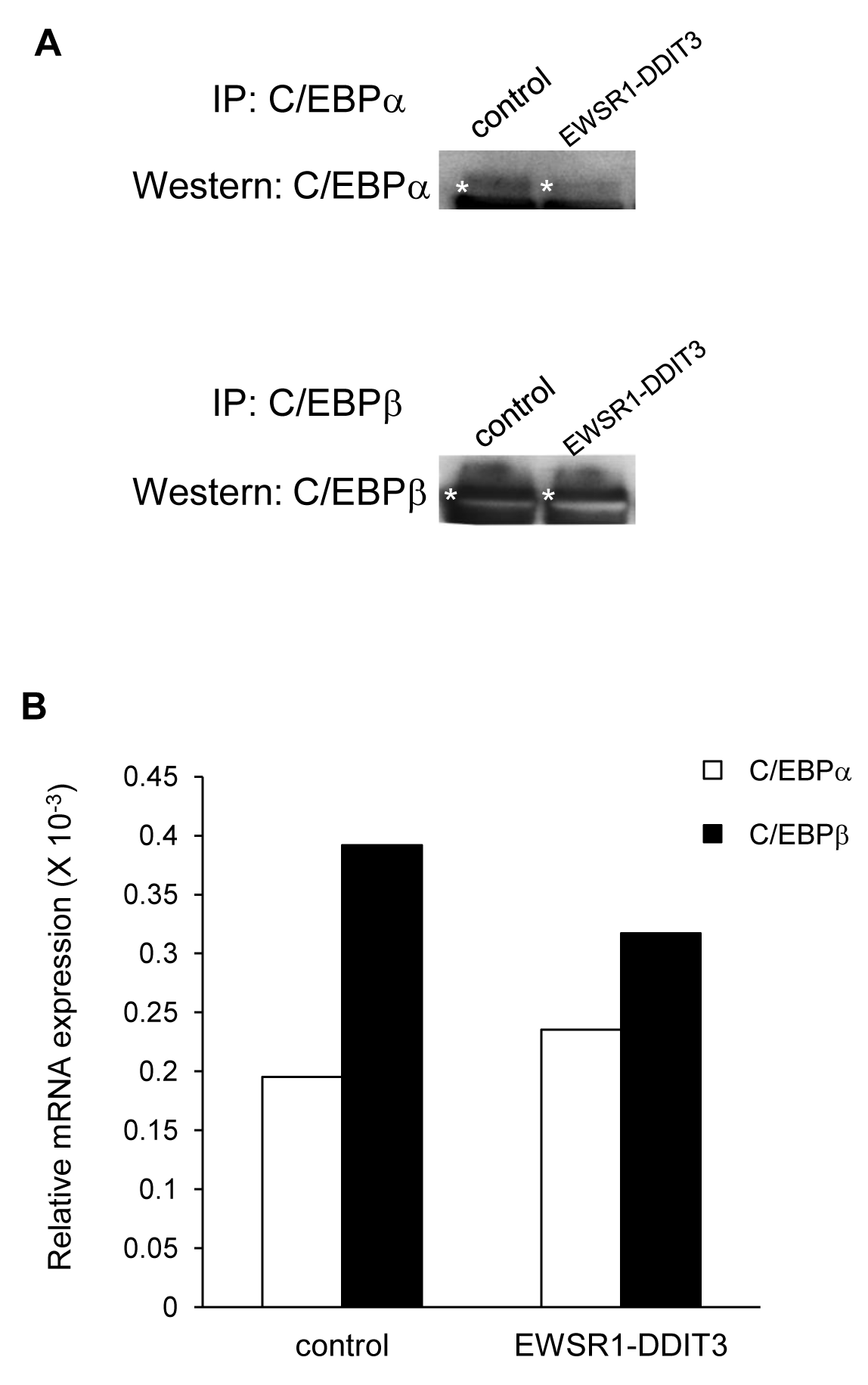

Supplement: Figure S2 — MV4 control or EWSR1-DDIT3. (A) The lysate from C3H10T1/2 cells transfected with pFLAG-CMV4 control or EWSR1-DDIT3 was immunoprecipitated (IP) with an anti-C/EBPα antibody or an anti-C/EBPβ antibody. IP samples were electrophoresed and blotted with an anti-C/EBPα antibody or an anti-C/EBPβ antibody. Each protein band is indicated by an asterisk (*). (B) Real-time quantitative PCR assay for the endogenous mRNA levels of C/EBPα and C/EBPβ in C3H10T1/2 cells transfected with pFLAG-CMV4 control or EWSR1-DDIT3. Each mRNA expression level was normalized to that of β-actin. Similar results were obtained in three independent experiments. (TIF) [file pone.0036682.s002.tif]

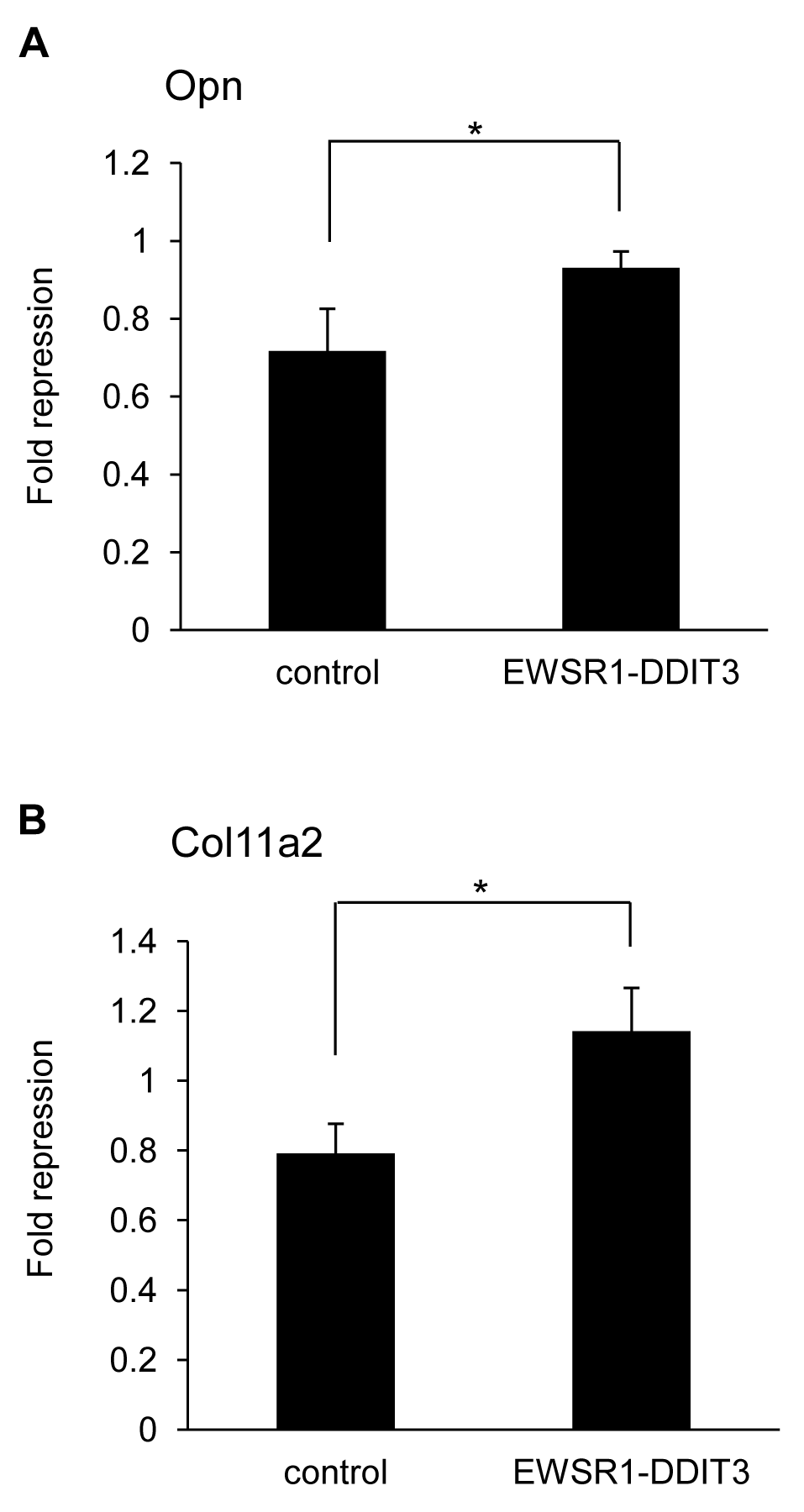

Supplement: Figure S3 — Repression of Opn or Col11a2 promoter activity by anacardic acid (AA), a small molecule compound which inhibits histone acetyltransferase (HAT) activity of p300 and PCAF, was significantly attenuated by overexpressing EWSR1-DDIT3. Effect of AA on Opn (A) and Col11a2 (B) promoter activities. C3H10T1/2 cells in duplicate plates were cotransfected with each promoter reporter construct plus EWSR1-DDIT3 expression vector. Cells in one plate were assayed for luciferase activity 24 h after treatment with AA (30 µM) and compared with the cells from the other plate that were not treated with AA. Luciferase activities from AA-treated cells relative to those from AA-untreated cells are shown as fold repression. Experiments in duplicate were repeated at least three times, and the results are shown as averages ± SE. An asterisk (*) indicates statistical significance (p<0.05) calculated by unpaired t-test, with p values of 0.0336 for Opn and 0.0401 for Col11a2. (TIF) [file pone.0036682.s003.tif]

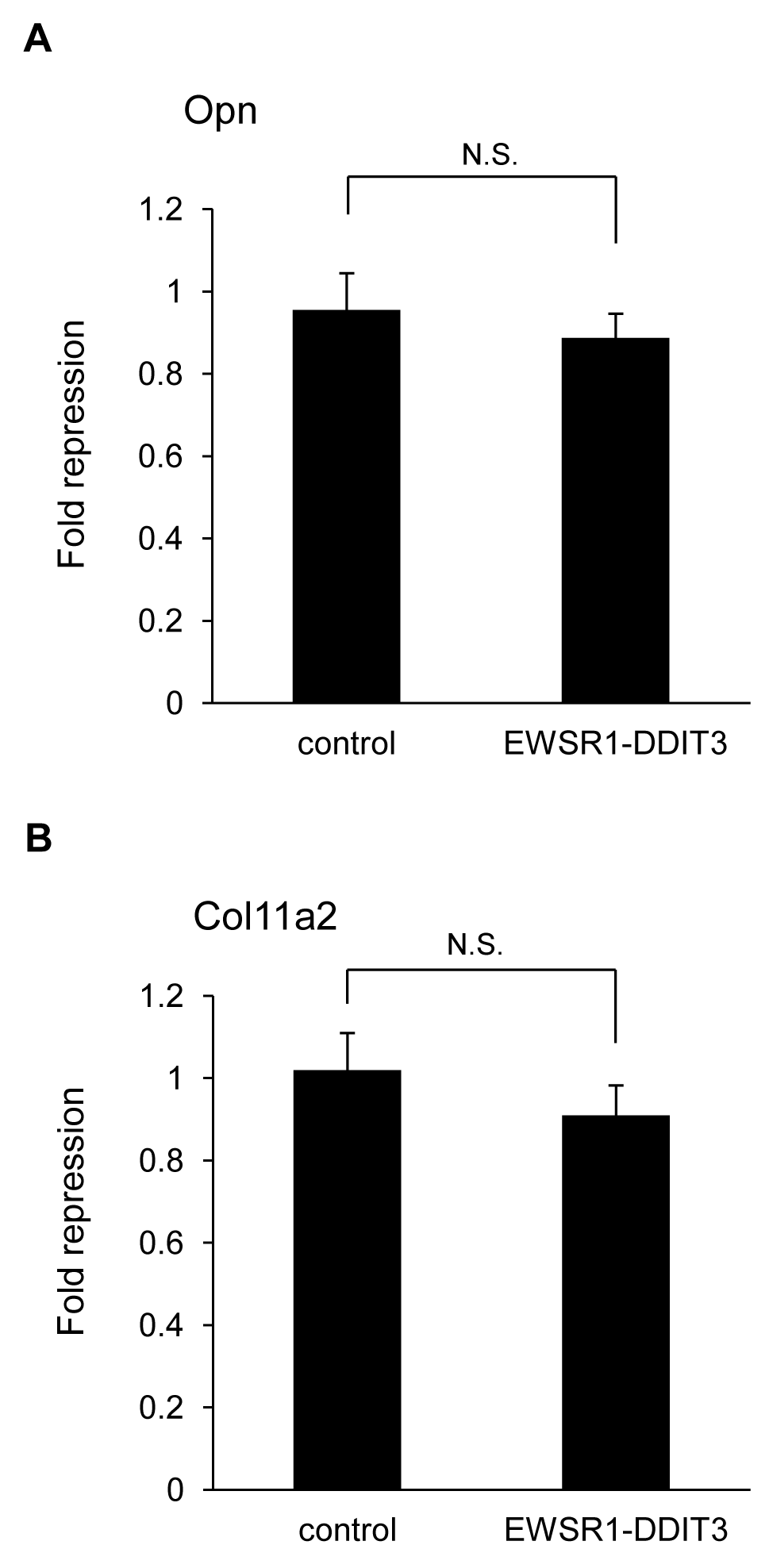

Supplement: Figure S4 — Temozolomide (TMZ), a DNA methylating chemotherapeutic drug, did not significantly influenced on the Opn and Col11a2 promoter activities. Effect of TMZ on Opn (A) and Col11a2 (B) promoter activities. C3H10T1/2 cells in duplicate plates were cotransfected with each promoter reporter constructs plus EWSR1-DDIT3 expression vector. Cells in one plate were assayed for luciferase activity 44 h after treatment with TMZ (50 µM) and compared with the cells from the other plate that were not treated with TMZ. Luciferase activities from TMZ-treated cells relative to those from TMZ-untreated cells are shown as fold repression. Experiments in duplicate were repeated at least three times, and the results are shown as averages ± SE. N.S., not significant. (TIF) [file pone.0036682.s004.tif]
